# Avian eggshell thickness in relation to egg morphometrics, embryonic development, and mercury contamination

**DOI:** 10.1002/ece3.6570

**Published:** 2020-07-20

**Authors:** Sarah H. Peterson, Joshua T. Ackerman, Mark P. Herzog, Matthew S. Toney, Breanne Cooney, C. Alex Hartman

**Affiliations:** ^1^ U.S. Geological Survey, Western Ecological Research Center Dixon Field Station Dixon CA USA

**Keywords:** avian, body mass, egg length, egg measurements, egg width, fresh wet weight

## Abstract

Eggshell thickness is important for physiological, ecological, and ecotoxicological studies on birds; however, empirical eggshell thickness measurements for many species and regions are limited. We measured eggshell thickness at the equator and the egg poles for 12 avian species and related eggshell thickness to egg morphometrics, embryonic development, egg status, and mercury contamination. Within an egg, eggshells were approximately 5.1% thicker at the equator than the sharp pole of the egg, although this difference varied among species (0.6%–9.8%). Within Forster's tern (*Sterna forsteri*), where eggshell thickness was measured at 5 equally spaced positions along the longitude of the egg, eggshell thickness changed more rapidly near the sharp pole of the egg compared to near the blunt pole of the egg. Within species, eggshell thickness was related to egg width and egg volume for six of the 12 species but was not related to egg length for any species. Among species, mean eggshell thickness was strongly related to species mean egg width, egg length, egg volume, and bird body mass, although species mean body mass was the strongest predictor of species mean eggshell thickness. Using three species (American avocet [*Recurvirostra americana*], black‐necked stilt [*Himantopus mexicanus*], and Forster's tern), whose nests were carefully monitored, eggshell thickness (including the eggshell membrane) did not differ among viable, naturally abandoned, dead, or failed‐to‐hatch eggs; was not related to total mercury concentrations of the egg content; and did not decrease with embryonic age. Our study also provides a review of all existing eggshell thickness data for these 12 species.

## Graphical abstract

We measured eggshell thickness at the egg equator and the egg poles for 12 avian species, and related eggshell thickness to egg morphometrics, embryonic development, egg status, and mercury contamination. Our study also provides a review of all existing eggshell thickness data for these 12 species.

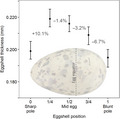

## 1 INTRODUCTION

Egg morphology has been the subject of many physiological (Ar, Paganelli, Reeves, Greene, & Rahn, 1974; Rahn & Ar, 1980; Rahn, Parisi, & Paganelli, 1982), ecological (Maurer, Russell, & Cassey, 2010; Rahn & Paganelli, 1988, 1989), and ecotoxicological studies (Cooke, 1973; Hickey & Anderson, 1968; Ratcliffe, 1970), yet empirical data for eggshell thickness are limited for many avian species and regions. For example, eggshell thickness can influence egg physiology, specifically gas exchange, because the diffusive properties of gas through pores in the eggshell relate to the ratio of pore length (eggshell thickness) to pore radius (Rahn, Paganelli, & Ar, 1987). Ecologically, eggshell thickness may vary as a result of factors including maternal age (Massaro & Davis, 2006), egg‐laying order (Castilla, Herrel, Robles, Malone, & Negro, 2010), egg mass (Castilla, Herrel, et al., 2010), and egg pigmentation (Gosler, Higham, & Reynolds, 2005). Some of these factors vary enough within and among clutches that they could cause marked differences in eggshell thicknesses among eggs; however, it is unclear whether this variation would result in an eggshell thickness that could influence whether or not an egg would hatch. Eggshell thickness also is an important egg measurement for ecotoxicological studies because it can be directly influenced by contaminant exposure (Cooke, 1973; Hickey & Anderson, 1968) and eggshell thickness is a necessary component in the accurate calculation of egg contaminant concentrations (Herzog, Ackerman, Eagles‐Smith, & Hartman, 2016).

In birds, embryonic development can influence the thickness of the eggshell, with the calcite eggshell thinning as the embryo develops (Ancel & Girard, 1992; Balkan, Karakaş, & Biricik, 2006; Castilla, Herrel, et al., 2010; Finnlund, Hissa, Koivusaari, Merila, & Nuuja, 1985; Orłowski & Hałupka, 2015; Orłowski, Hałupka, Klimczuk, & Sztwiertnia, 2016; Orłowski, Merta, et al., 2019; Santolo, Byron, & Ohlendorf, 2016). Consequently, hatched eggs have thinner eggshells than freshly laid eggs simply due to embryonic development, as calcium is mobilized from the mammillary tips within the calcite eggshell and into the interior of the egg for embryonic growth (Karlsson & Lilja, 2008; Orłowski & Hałupka, 2015). However, as the calcite eggshell thins during development, eggshell membranes may increase in thickness (Castilla, Van Dongen, et al., 2010) and become less tightly attached to the calcite eggshell (Finnlund et al., 1985). Consequently, the thickness of the combined eggshell and membrane may not change as a result of embryonic development, and most studies examining eggshell thickness in wild birds include the membrane in the measurement of the eggshell.

Contaminant exposure, particularly to organochlorine pesticides, can decrease eggshell thickness and influence egg survival (Cooke, 1973; Hickey & Anderson, 1968). It is possible that other environmental contaminants, such as mercury, may influence eggshell thickness as well, but only a few studies have examined this in bird eggs (Blus, Heath, Gish, Belisle, & Prouty, 1971; Hargreaves, Whiteside, & Gilchrist, 2011; Heinz, 1979; King, Custer, & Quinn, 1991; Lundholm, 1995; Rodriguez‐Navarro, Gaines, Romanek, & Masson, 2002; Stoewsand, Anderson, Gutenmann, Bache, & Lisk, 1971).

In ecotoxicological studies, eggshell thickness influences the estimation of an egg's contaminant concentration (Herzog et al., 2016). The ideal reporting metric of contaminant concentrations in avian eggs is the calculation of fresh wet weight (fww) of the egg (Ackerman, Herzog, & Schwarzbach, 2013), a calculation that typically uses estimates of egg density, egg volume, and fresh egg mass. Estimating these measurements without removing the eggshell can result in a 6%–13% underestimate of egg contaminant concentrations (Herzog et al., 2016). Consequently, the calculation of contaminant concentrations in egg contents can be improved by estimating and subsequently excluding the thickness of the eggshell (Herzog et al., 2016). The common allometric equations to estimate eggshell thickness use egg length, egg width, eggshell mass, or whole egg mass (Ar et al., 1974; Khurshid, Farooq, Durrani, Sarbiland, & Chand, 2003; Maurer et al., 2010; Morrison & Kiff, 1979; Osborne & Winters, 1977; Ratcliffe, 1970) and are derived from large, multispecies datasets, although their accuracy has not been well validated for individual eggs (Ancel & Girard, 1992; Maurer, Portugal, & Cassey, 2012). Additionally, eggshell thickness may be estimated using bird body mass (Birchard & Deeming, 2009). The main equation to predict eggshell thickness from egg mass was derived from Schönwetter (1960–1992); this equation can be misused because it was derived using estimates of eggshell thickness from other equations and was not empirically based (Maurer et al., 2012). Furthermore, egg mass decreases by as much as 15% during embryonic development (Brown, 1976; Drent, 1970; Westerskov, 1950) and egg mass can also decrease as a result of desiccation from environmental exposure. Therefore, predictive equations based on egg mass (Ar & Rahn, 1985; Osborne & Winters, 1977; Rahn & Paganelli, 1989) will only be accurate for freshly laid eggs because the relationship between eggshell thickness and egg mass changes after the time point when the egg was laid. Thus, eggshell measurements are needed within and among species to test and improve upon allometric relationships for estimating eggshell thickness (using egg morphometrics or bird mass) that are not based on egg mass and are accurate for individual species.

We used 12 avian species to provide empirical eggshell thickness measurements in relation to egg morphometrics, embryonic development, egg status at the time of collection, and mercury contamination. The methodology we used provided more precise and repeatable eggshell thickness measurements than prior studies that used analog micrometers (Santolo, 2018), and these eggshell thicknesses can be applied in other ecological, physiological, and toxicological studies. Specifically, we examined the following: (a) eggshell thickness at multiple positions on the egg; (b) the relationship between eggshell thickness and egg morphometrics (length, width, and volume) both within and among species, as well as the relationship between species mean eggshell thickness and species mean bird body mass; (c) whether eggshell thickness decreases with embryonic development; (d) whether there are differences in eggshell thickness related to the egg status at the time of collection (normally developing eggs, eggs naturally abandoned by parents, dead embryos in eggs from nests where no sibling eggs hatched, and dead embryos in eggs from nests where sibling eggs hatched); and (e) whether eggshell thickness is related to egg content mercury concentrations. Eggshell thickness in relationship to mercury contamination was chosen because few studies have examined the effects of mercury on eggshell thickness and mercury concentrations were analyzed for related contaminant studies.

## 2 METHODS

### 2.1 Sample collection

We salvaged and collected eggs from 12 avian species, representing 6 families from 4 orders (Table 1), as part of related contaminant studies during 2014–2018 (Peterson & Ackerman, 2020). Eggs of 11 species were from multiple sites within San Francisco Bay and the Central Valley in California (USA), some Caspian tern (*Hydroprogne caspia*) eggs were from the Potholes Reservoir in Washington State (USA), and wood duck (*Aix sponsa*) eggs were from Fallon, Nevada (USA). In the field, eggs were placed in egg cartons and kept in small coolers with wet ice until they were transported back to the laboratory. Eggs were stored in a refrigerator (2°C) until processing.

**TABLE 1:** Eggshell thickness was measured for 12 avian species from 4 orders and 6 families

Order	Family	Common name	Scientific name	Mean female body mass (g)
Anseriformes	Anatidae	Mallard	*Anas platyrhynchos*	1,095
	Anatidae	Wood duck	*Aix sponsa*	647
Charadriiformes	Charadriidae	Western snowy plover	*Charadrius nivosus nivosus*	42
	Laridae	Black skimmer	*Rynchops niger*	254
	Laridae	California gull	*Larus californicus*	599
	Laridae	California least tern	*Sternula antillarum browni* ^a^	44
	Laridae	Caspian tern	*Hydroprogne caspia*	670
	Laridae	Forster's tern	*Sterna forsteri*	136
	Recurvirostridae	American avocet	*Recurvirostra americana*	340
	Recurvirostridae	Black‐necked stilt	*Himantopus mexicanaus*	169
Pelecaniformes	Ardeidae	Great egret	*Ardea alba*	883
Suliformes	Phalacrocoracidae	Double‐crested cormorant	*Phalacrocorax auritus albociliatus* ^b^	1,831

Note

Mean female body mass was obtained from published studies (Ackerman, Hartman, et al., 2013; Ackerman et al., 2008; Bluso et al., 2006; Delnicki & Reinecke, 1986; Dunning, 2008; Herring et al., 2008, 2010b; Page et al., 2009; Robinson et al., 1999).

^a^ Available body mass measurements were for the subspecies *Sternula antillarum athalassos*.

^b^ Available body mass measurements were for the subspecies *Phalacrocorax auritus auritus*.

### 2.2 Eggshell processing

First, the exterior of each egg was cleaned with deionized water, swabbed with isopropyl alcohol, rinsed with deionized water, and allowed to dry. Before egg dissection, length (±0.01 mm) and width (±0.01 mm) were measured using digital calipers (Mitutoyo, Aurora, Illinois, USA) and whole egg mass (±0.01 g) was obtained with a digital balance (Ohaus Adventurer™ Pro AV212, Ohaus Corporation). We then cut an approximately 15 mm diameter circle at the blunt end of each egg using stainless‐steel scissors, removed the blunt end of the eggshell, and transferred the egg contents into a sterile polypropylene jar. The blunt pole of the eggshell was removed and discarded during egg processing for some eggs, prior to the development of this specific study. Embryos were aged to the nearest whole day (Ackerman & Eagles‐Smith, 2010), and the egg contents were prepared for determination of mercury (Ackerman, Eagles‐Smith, Herzog, & Hartman, 2016). Most eggs (68.8%) were identified as fertile and aged to at least 1 day in incubation (mean 7.8 ± 5.2 days; interquartile range 4–11 days; range 1–27 days). Additionally, 3.2% were identified as fresh and fertile (day 0 of incubation). The remaining eggs were either infertile or embryonic age could not be determined. After egg dissection, eggshells were stored in a freezer at −20°C.

Prior to processing of eggshells and measurement of eggshell thickness, eggshells were removed from the freezer and allowed to warm to room temperature. The outside of the eggshell was reexamined to determine whether there was any remaining exogenous material that needed to be removed. Then, we rinsed the inside of eggshells with a mild detergent (Alconox) and used a cotton swab to wipe out the inside. If necessary, a small stainless‐steel spatula was gently used to dislodge any contents adhered to the inside of the eggshell that could not be dislodged with a cotton swab. After any remaining egg contents were dislodged, the inside was rinsed multiple times with deionized water. Eggshell membranes were not removed. We recorded the condition of the ultrathin outermost eggshell membrane, closest to the egg contents (Simkiss, 1961), because that membrane occasionally becomes detached from the rest of the eggshell during dissection and it is almost always absent from the blunt pole as it peels away from the eggshell in the blunt pole region as the air cell expands during embryonic development. The main inner eggshell membrane (Simkiss, 1961) was present in all eggshells. Once cleaned, eggshells were placed in a drying oven for 24 hr at 40°C and stored in a desiccator until they were measured.

### 2.3 Eggshell thickness measurements

**FIGURE 1:**
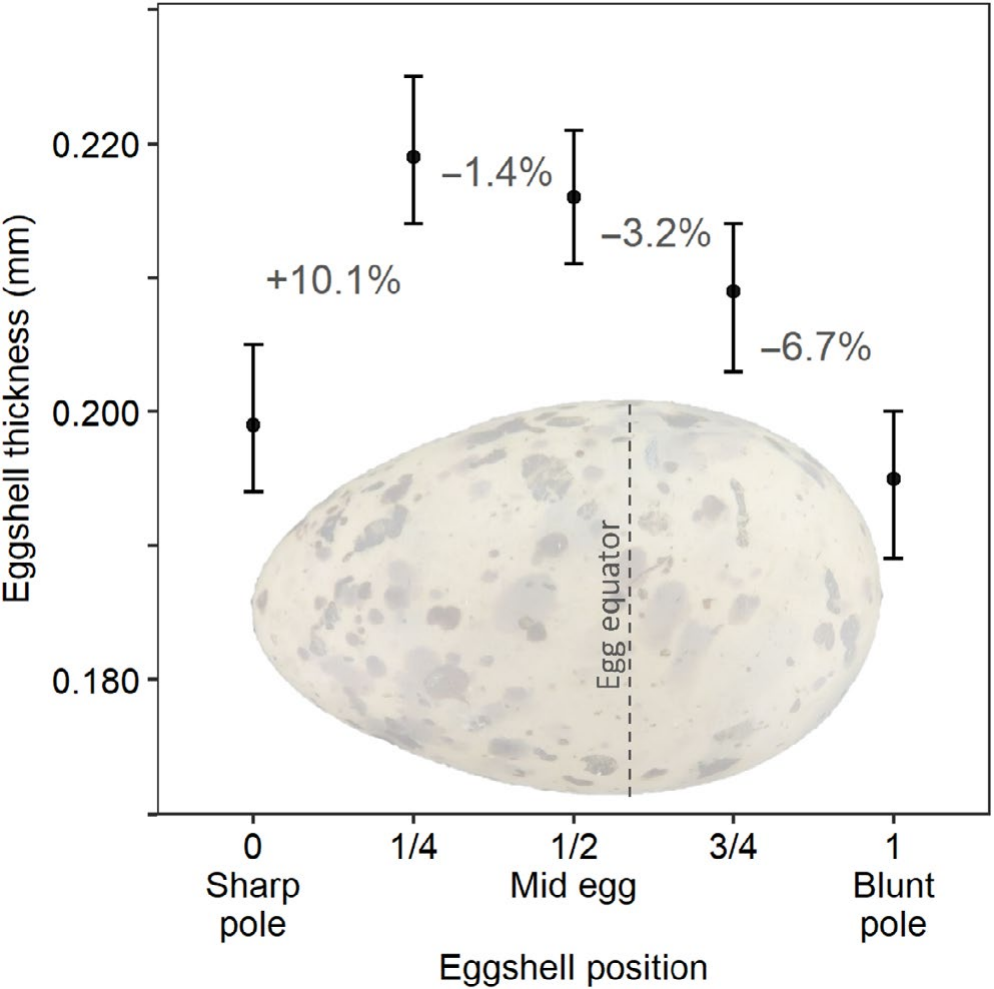
Least squares mean eggshell thickness (±95% CI) at 5 different equally spaced positions on Forster's tern eggshells (*n* = 40). Percentages represent the percent thicker or thinner each location is relative to the measurement location directly to the left, moving from the sharp pole toward the blunt pole. Note that the ½ egg was measured at the midpoint of the egg and not the widest part of the egg (which is called the egg equator; shown with a dashed line) that is typically closer to the blunt pole than the sharp pole

We measured eggshell thickness at 3 positions on each eggshell when possible: equator, sharp pole, and blunt pole (Figure 1). We measured eggshell thickness using a Magna‐Mike^®^ 8600 Hall effect thickness gauge (Olympus Scientific Solutions Americas Corporation) with a 1.58 mm magnetic measurement ball. We measured the minimum thickness of the eggshell and membrane as the ball was rolled across the inside of the eggshell at three measurement positions: the equator, the sharp pole, and the blunt pole, following the methods of Santolo (2018). At the equator measuring position, we slowly rotated the egg 3–5 times over the measurement ball to make sure the entire equator was sampled. At the sharp and blunt pole measuring positions, the measurement ball was rolled around in a small circle to capture the entire end of the eggshell. Our method measured across maculated (pigment spots) and plain sections of eggshells. Because some studies showed differences in eggshell thickness between pigmented and unpigmented sections (Gosler et al., 2005), our method captured the thinnest spot at that measurement position on the eggshell, which may have represented a pigmented section. If only a portion of the eggshell was intact at the equator, we measured as much of the eggshell as possible and recorded the percent of the eggshell area that was sampled. We excluded measurements from sections of eggshell that had mold on them and any eggshells where the main inner eggshell membrane was removed or was visibly separating from the calcite portion of the eggshell. The thickness gauge was calibrated at the start of every day of measurement and any time when the machine was inactive for more than 1 hr.

To examine more specifically whether and how eggshell thickness changed longitudinally from the sharp to the blunt pole of the eggshell, we conducted a separate experiment where we measured eggshell thickness at 5 positions (Figure 1) on a subset of Forster's tern (*Sterna forsteri*) eggshells (*n* = 40) where the blunt pole eggshell piece was retained after egg dissection. The sharp pole and blunt pole were measured in the same way as described above, but we also measured eggshell thickness at 3 additional and equally spaced regions on the egg at the ¼ egg (i.e., halfway between the sharp pole and the midpoint of the egg), the ½ egg (i.e., the midpoint of the egg), and the ¾ egg (i.e., halfway between the midpoint of the egg and the blunt pole). The ½ egg was measured at the midpoint of the egg and not the egg equator, which is defined as the widest part of the egg and typically the equator is closer to the blunt pole than the sharp pole of the egg (Figure 1). Each of the 5 positions on an individual eggshell was measured within the same calibration period (<15 min). We used the same protocol described above, turning the eggshell 3–5 times over the measurement ball around each longitudinal section.

### 2.4 Mercury determination

Eggs were analyzed for total mercury (THg) using a Nippon Instruments MA‐3000 Direct Mercury Analyzer (Nippon Instruments Corporation) at the U.S. Geological Survey Dixon Field Station Environmental Mercury Laboratory, following U.S. Environmental Protection Agency Method 7473 (U.S. Environmental Protection Agency, 2000). This method uses an integrated sequence of drying, thermal decomposition, catalytic conversion, and then amalgamation, followed by atomic absorption spectroscopy. Dried and homogenized egg aliquots were weighed to the nearest 0.00001 g prior to analysis (Mettler Toledo XS105). Egg THg concentrations (µg/g) are reported as fresh wet weight (fww), following Ackerman, Herzog, et al. (2013) as modified by Herzog et al. (2016) to exclude the thickness of the measured eggshell from each egg.

Standard measures of quality assurance were used, including determination of THg concentrations in certified reference materials (DORM‐4, DOLT‐4, DOLT‐5, IAEA‐407, TORT‐3; National Research Council Canada and International Atomic Energy Agency), determination of THg concentrations in internal laboratory reference materials, matrix spikes, continuing calibration verifications, duplicates, and system and method blanks in each run of samples. The mean (±*SD*) relative percent difference of duplicate samples was 2.7 ± 2.8% (*n* = 290). Recoveries were 100.2 ± 2.8% (*n* = 368) for certified reference materials, 99.9 ± 2.1% (*n* = 328) for calibration verifications, and 100.6 ± 3.5% (*n* = 322) for matrix spikes.

### 2.5 Statistical analyses

We examined the influence of species, measurement position, egg morphometrics (egg length, egg width, and egg volume) and bird body mass, embryo age, egg status at the time of collection, and egg THg content on eggshell thickness using a combination of weighted regression, mixed effects linear models, and general linear models. All analyses were performed in the statistical program R (R Core Team, 2019).

#### 2.5.1 Eggshell thickness among species and within individual eggs

We examined whether there were differences in eggshell thickness within and among species at specific egg measurement positions within individual eggshells. First, we compared the eggshell thickness values from 5 positions in the detailed study of Forster's tern eggs with a linear mixed effects model using the lme4 package (Bates et al., 2015). In the model, we included measurement position as a fixed effect and eggshell identification as a random effect. We used the Kenward–Roger approximation for degrees of freedom and tested for significance with *F* tests generated from the afex package (Singmann, Bolker, & Westfall, 2015). We examined differences in model‐generated least squares mean eggshell thicknesses among the five eggshell measurement positions with a Tukey honest significant difference adjustment.

Using data from multiple species (*n* = 12), we ran two linear mixed effects models with species, measurement position on the eggshell, and a species × measurement position interaction as fixed effects and eggshell identification nested within nest identification as random effects. The first model included eggshells with paired equator and sharp pole measurements, and the second model included eggshells that had an equator, sharp pole, and blunt pole measurement.

We examined whether there was a consistent difference in eggshell thickness between the sharp pole and the equator within and among species using two approaches. First, we examined the relationship between the sharp pole and the equator using a general linear model with individual eggshell thickness measurements and an equator eggshell thickness × species interaction. Second, we examined the linear relationship among species, using a weighted regression on species mean values of each measurement. We calculated weights as the natural log of the sample size to reduce the weight on higher sample sizes such that species with more samples were weighted only slightly more than species with fewer samples. We then calculated residuals for all individual eggshell measurements from the regression equation generated using the species means. Finally, we used the mean and 95% CI of the residuals to determine whether the residuals for each species fell above, included, or were below zero. If the 95% CI of the residuals for a species included zero, that would suggest that the mean eggshell thickness at the sharp pole for that species was within the range of what would be expected based on the eggshell thickness at the equator. Conversely, if the 95% CI of the residuals for a species was entirely above zero, that would suggest that the eggshell thicknesses at the sharp pole for that species were thicker than would be expected based on the eggshell thickness at the equator. If the 95% CI of the residuals for a species was entirely below zero, that would suggest that the eggshell thicknesses at the sharp pole for that species were thinner than would be expected based on the eggshell thickness at the equator.

#### 2.5.2 Eggshell thickness versus egg morphometrics and bird body mass

We quantified the relationship between eggshell thickness at the equator and egg morphometrics (egg length, width, and volume) within and among species. First, we examined the relationship between the eggshell thickness at the equator and either the egg length, egg width, or egg volume (in separate models), using a general linear model with equator eggshell thickness measurements for individual eggs and an equator eggshell thickness × egg morphometric measurement interaction. We then ran individual models for each species with more than 10 samples. Egg volume was calculated using an egg shape coefficient (*K_v_*), egg length, and egg width (egg volume = *K_v_* × egg length × egg width^2^; Hoyt, 1979). Second, to qualitatively compare the relationship between the eggshell thickness at the equator and egg morphometrics within versus among species, we quantified the linear relationship among species (length, width, and volume in separate models), using a weighted regression on species mean values, with weights calculated as the natural log of the sample size to reduce the weight on higher sample sizes such that species with more samples were weighted only slightly more than species with fewer samples. We then calculated residuals for all individual eggshells from the regression equation generated using species means and used these residuals to determine whether the mean residual value for each species fell above, included, or was below zero, using the mean and 95% CI for each species. If the 95% CI of the residuals for a species included zero, that would suggest that the mean eggshell thickness at the equator pole for that species was within the range of what would be expected based on the egg length or width. Conversely, if the 95% CI of the residuals for a species was entirely above zero, that would suggest that the eggshell thickness at the equator for that species was thicker than would be expected based on the egg length or width. If the 95% CI of the residuals for a species was entirely below zero, that would suggest that the eggshell thickness at the equator for that species was thinner than would be expected based on the egg morphometric measurements.

We also quantified the relationship between species mean eggshell thickness at the equator and species mean bird body mass. For bird masses, we used published mean female body masses (Ackerman, Hartman, et al., 2013; Ackerman, Takekawa, Bluso, Yee, & Eagles‐Smith, 2008; Bluso, Ackerman, Takekawa, & Yee, 2006; Delnicki & Reinecke, 1986; Dunning, 2008; Herring, Ackerman, Eagles‐Smith, & Takekawa, 2010; Herring, Gawlik, & Beerens, 2008; Page, Stenzel, Warriner, Warriner, & Paton, 2009; Robinson, Reed, Skorupa, & Oring, 1999; Table 1). We transformed bird mass (log_10_) prior to analysis because we did not expect bird body mass to scale linearly with eggshell thickness (Birchard & Deeming, 2009). We used AICc (corrected for small sample sizes) to compare regression models with different predictors of eggshell thickness.

#### 2.5.3 Eggshell thickness versus embryo age

We examined whether eggshell thickness measured at the equator and sharp pole decreased with embryonic development, using a subset of normally developing eggs of American avocet (*Recurvirostra americana*), black‐necked stilt (*Himantopus mexicanus*), and Forster's tern that were collected during weekly nest monitoring. We used a general linear model with fixed effects for species, embryo age (in days), and a species × embryo age interaction. We did not include nest identification as a random effect because we had only 1 normally developing egg from each nest.

#### 2.5.4 Eggshell thickness versus egg status

For a subset of American avocet, black‐necked stilt, and Forster's tern eggs that were sampled during weekly nest monitoring visits, the status of each egg was categorized upon collection as active, abandoned, dead, or failed to hatch (Herring, Ackerman, & Eagles‐Smith, 2010). Active eggs were normally progressing in nests that were actively being incubated, whereas abandoned eggs were from nests where the parents naturally had abandoned the nest. Eggs classified as dead contained dead embryos and had stopped progressing normally in nests while they were still being incubated and no sibling eggs in the clutch hatched. Failed‐to‐hatch eggs contained dead embryos and also did not hatch but were from nests where other sibling eggs in the clutch successfully hatched. We ran two separate mixed effects linear models to compare eggshell thickness at either the equator or the sharp pole with egg status, species, embryo age, an egg status × species interaction, and nest identification as a random effect. Nest identification was included as a random effect, because some nests with dead, abandoned, or failed‐to‐hatch eggs had multiple eggs salvaged.

#### 2.5.5 Eggshell thickness versus egg content mercury

To examine whether eggshell thickness was related to MeHg exposure, we used the subset of normally progressing American avocet, black‐necked stilt, and Forster's tern eggs. We determined THg concentration in the egg as a proxy for MeHg since 96% of the Hg in bird eggs is in the MeHg form (Ackerman, Herzog, et al., 2013). We used two general linear models, one for the equator eggshell thickness and one for the sharp pole eggshell thickness, with fixed effects for the egg content THg concentration (fww), species, embryo age, and an egg content THg concentration × species interaction. We did not include nest identification as a random effect because only 1 normally developing egg in this dataset was from each nest.

## 3 RESULTS

### 3.1 Eggshell thickness among species

**FIGURE 2:**
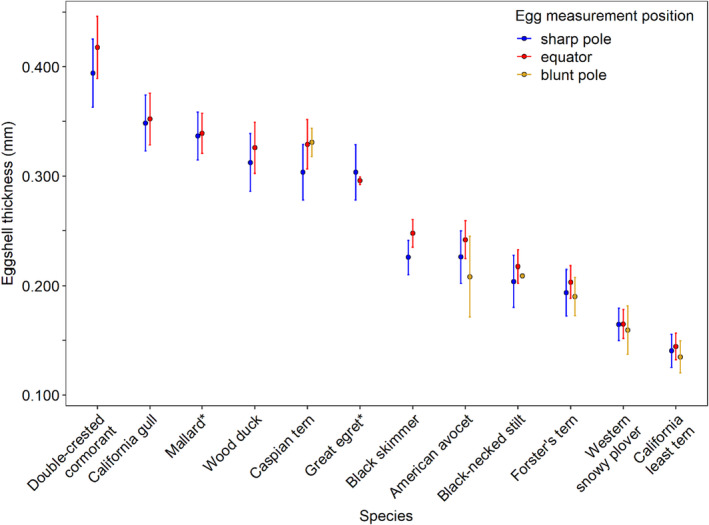
Arithmetic mean eggshell thickness (±*SD*) at the egg equator, sharp pole, and blunt pole for 12 different avian species (raw data). Asterisks indicate species with <10 eggs measured. Refer to Table 2 for sample sizes and egg length and egg width measurements

We observed a 190% difference in mean eggshell thickness at the equator between the species with the thinnest eggshells (California least tern [*Sternula antillarum browni*]: 0.144 mm) and the thickest eggshells (double‐crested cormorant [*Phalacrocorax auratus albociliatus*]: 0.418 mm; Figure 2). Similarly, we observed a 181% difference in mean eggshell thickness at the sharp pole between the species with the thinnest eggshells (California least tern: 0.140 mm) and the thickest eggshells (double‐crested cormorant: 0.394 mm). The range in observed eggshell thickness varied within species but was far more extensive among species; 92.6% of the variance in eggshell thickness at the equator occurred among species compared to 7.4% within species. Similarly, 87.0% of the variance in eggshell thickness at the sharp pole occurred among species compared to 13.0% within species.

### 3.2 Eggshell thickness varies within individual eggs

#### 3.2.1 Eggshell thickness at 5 equally spaced positions on the egg

We began with a detailed study of eggshell thickness among 5 equally spaced positions on Forster's tern eggs (*n* = 40). Eggshells were thickest at the ¼ egg and ½ egg and thinnest at the poles (*F*_4,156.0_ = 31.96,* p* < .001; Figure 1). The increase in eggshell thickness was greater between the sharp pole and the ¼ egg than between the blunt pole and the ¾ egg (Figure 1). Furthermore, average eggshell thickness was similar at the ¼ egg and the ½ egg (*t* = 1.29, *p* = .70), whereas eggshell thickness at the ¾ egg was 3.2% thinner than the ½ egg (*t* = 2.70, *p* = .059). For this set of eggshells, the ½ egg was 10.8% thicker than the blunt pole (*t* = 8.06, *p* < .001) and 8.5% thicker than the sharp pole (*t* = 6.32, *p* < .001), and the sharp pole was similar in thickness to the blunt pole (*t* = 1.74, *p* = .42).

#### 3.2.2 Eggshell thickness at the equator and poles

When we considered all 12 species, avian eggshells generally were thickest at the equator and thinner at the sharp and blunt poles. On average, eggshells were 5.1% thicker at the equator than the sharp pole when there were at least 10 eggshells measured from a species (*n* = 10 species; Table 2), although mean differences between eggshell thickness at the equator and sharp pole within a species varied from 0.6% to 9.8% (*F*_11,2500.0_ = 19.46, *p* < .001; Figure 2). Western snowy plover (*Charadrius nivosus nivosus*) was the only species with ≥10 eggshells measured where there was no detectable difference between the eggshell thickness at the equator and the sharp pole (*t* = 0.19, *p* = .85, 0.6% difference in mean values; all other species *t* ≥ 2.32, *p* ≤ .02). Black skimmer (*Rynchops niger*) had the greatest difference between the eggshell thickness at the equator and the sharp pole (9.8%), followed by Caspian tern (8.3%), American avocet (7.1%), double‐crested cormorant (6.1%), black‐necked stilt (5.9%), Forster's tern (4.6%), wood duck (4.5%), California least tern (2.9%), and California gull (*Larus californicus*; 1.1%).

**TABLE 2:** Sample size (*n*) for egg morphometric data (egg length and egg width) salvaged and collected from 12 avian species between 2014 and 2018 throughout western North America

Species	*n*	Egg length (mm) ± *SD* (range)	Egg width (mm) ± *SD* (range)	Equator *n*	Eggshell thickness at equator (mm) ± *SD* (range)	Sharp pole *n*	Eggshell thickness at sharp pole (mm) ± *SD* (range)	Blunt pole *n*	Eggshell thickness at blunt pole (mm) ± *SD* (range)
American avocet	844	49.19 ± 2.20 (41.31–56.34)	34.13 ± 1.11 (24.71–39.84)	843	0.242 ± 0.017 (0.156–0.297)	773	0.226 ± 0.024 (0.152–0.298)	6	0.208 ± 0.037 (0.146–0.252)
Black‐necked stilt	204	43.31 ± 1.76 (38.88–49.34)	30.99 ± 0.85 (27.86–33.16)	201	0.217 ± 0.015 (0.184–0.259)	179	0.204 ± 0.024 (0.143–0.261)	1	0.209
Black skimmer	11	47.78 ± 2.95 (42.90–51.91)	34.59 ± 1.06 (31.79–35.59)	11	0.247 ± 0.013 (0.229–0.273)	11	0.226 ± 0.015 (0.206–0.251)	0	NA
California gull	175	65.02 ± 2.87 (55.66–72.10)	45.29 ± 1.43 (41.22–48.60)	175	0.352 ± 0.024 (0.260–0.419)	162	0.348 ± 0.026 (0.275–0.419)	0	NA
California least tern	340	30.70 ± 1.28 (25.55–35.62)	22.43 ± 0.70 (20.30–24.34)	332	0.144 ± 0.012 (0.109–0.177)	249	0.140 ± 0.015 (0.095–0.201)	80	0.135 ± 0.015 (0.091–0.182)
Caspian tern	62	63.32 ± 2.44 (57.93–68.46)	43.76 ± 1.27 (40.53–47.02)	62	0.329 ± 0.022 (0.270–0.381)	60	0.303 ± 0.025 (0.217–0.353)	10	0.331 ± 0.013 (0.315–0.355)
Double‐crested cormorant	90	60.97 ± 2.61 (55.26–67.34)	39.02 ± 1.44 (34.04–42.22)	88	0.418 ± 0.029 (0.353–0.467)	89	0.394 ± 0.031 (0.305–0.473)	0	NA
Forster's tern	1,103	42.82 ± 1.78 (28.03–47.91)	30.11 ± 0.89 (21.75–33.54)	1,085	0.203 ± 0.015 (0.155–0.257)	946	0.194 ± 0.021 (0.116–0.307)	176	0.190 ± 0.018 (0.138–0.250)
Great egret	3	59.67 ± 1.50 (57.95–60.67)	40.47 ± 1.76 (38.51–41.92)	3	0.296 ± 0.004 (0.292–0.299)	3	0.303 ± 0.025 (0.281–0.331)	0	NA
Mallard	2	56.70 ± 1.98 (55.30–58.10)	40.94 ± 0.86 (40.33–41.55)	2	0.339 ± 0.018 (0.326–0.352)	2	0.337 ± 0.022 (0.321–0.352)	0	NA
Western snowy plover	35	30.67 ± 0.99 (28.81–32.98)	22.25 ± 0.89 (19.54–23.80)	34	0.165 ± 0.013 (0.133–0.191)	27	0.165 ± 0.015 (0.126–0.189)	9	0.159 ± 0.022 (0.122–0.186)
Wood duck	39	49.90 ± 2.07 (43.19–54.60)	38.04 ± 1.59 (32.64–40.59)	39	0.326 ± 0.023 (0.273–0.377)	38	0.312 ± 0.026 (0.255–0.377)	0	NA

Note

Sample size and raw data on minimum eggshell thickness (including the main inner eggshell membrane) measurements at the equator, sharp pole, and blunt pole. Sample sizes differ because egg morphometric measurements were not obtained for all eggs and some eggshells were not measured at all three egg positions. NA indicates when no measurements were taken at that measurement position for that species.

From the second model, when eggshell thickness was measured at the blunt pole, equator, and sharp pole on at least 10 eggshells from a species (*n* = 2 species: Forster's tern and California least tern), eggshells were, on average, 6.8% thicker at the equator than at the blunt pole (8.4% and 5.1%, respectively; all *t* ≥ 2.49, *p* ≤ .013). The comparison of eggshell thickness between the poles was less clear, with Forster's tern eggshells 5.3% thicker at the sharp pole (*t* = 6.94, *p* < .001) but no clear difference observed between eggshell thickness at the poles for California least tern (mean sharp pole 2.2% thicker; *t* = 0.93, *p* = .35).

#### 3.2.3 Relationship between sharp pole and equator eggshell thickness within species

**FIGURE 3:**
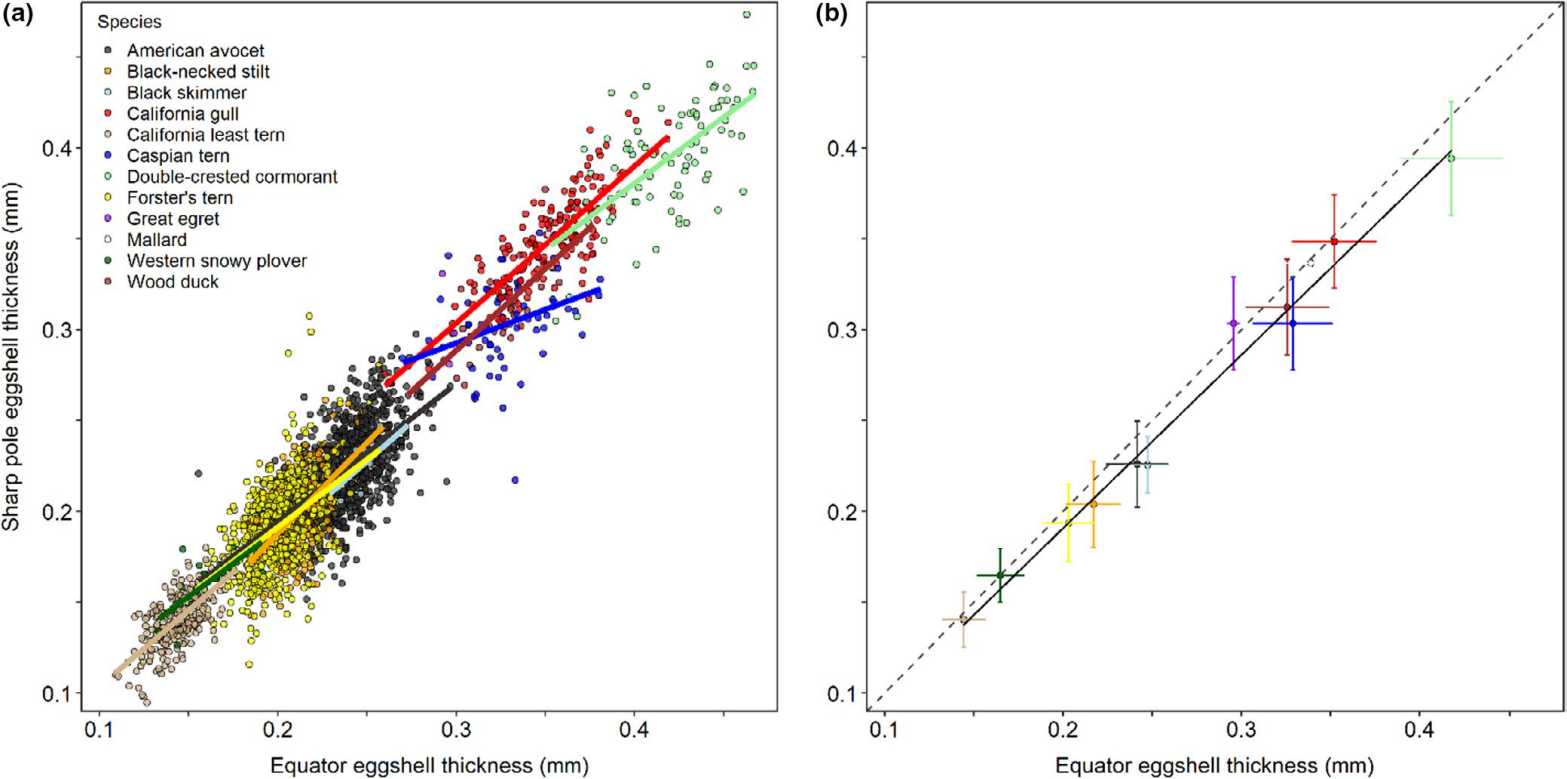
Eggshell thickness at the sharp pole was related to eggshell thickness at the equator during 2014–2018. (a) Individual eggshell measurements are shown with slopes for each species from individual species regressions. (b) Mean (± *SD*) eggshell thickness values (raw data) are shown for each species on top of the among‐species regression based on the species’ mean values. The solid line is the regression line (sharp pole thickness [mm] = (0.95300 × equator eggshell thickness [mm]) + 0.00029), shown for the range of the data means in the present study, and the dashed line indicates a theoretical 1:1 relationship

When individual eggshell thickness measurements (Table 2) and an equator eggshell thickness × species interaction were included in the model, eggshell thickness at the sharp pole was generally related to eggshell thickness at the equator, although there were some differences among species (*F*_11,2488_ = 2.52, *p* = .004). Within the global model, American avocet had a slope coefficient of 0.76 and, statistically, all but two species with more than 10 samples had similar slopes (all *t* ≤ 1.6, all *p ≥ *.11; slope range: 0.73–0.90). Caspian tern had a shallower slope coefficient than American avocet (*t* = 3.51, *p* < .001; slope = 0.37) and black‐necked stilt had a steeper slope coefficient than American avocet (*t* = 2.30, *p* = .02; slope = 1.00). When models were run individually, eggshell thickness at the sharp pole was significantly related to eggshell thickness at the equator for all 10 species (all *F* ≥ 7.05, all *p* ≤ .015, all *R* ^2^ ≥ .11; Figure 3a; Table 3).

**TABLE 3:** Slope and intercept values for individual species regressions between sharp pole eggshell thickness (mm) and equator eggshell thickness measurements (mm) for 10 individual species

Species	Slope	Intercept	*F_df_*	*R* ^2^	*p*‐Value
American avocet	0.75863	0.04271	*F*_1,769_ = 336.95	.30	<.001
Black‐necked stilt	1.00255	−0.01266	*F*_1,174_ = 93.23	.35	<.001
Black skimmer	0.85613	0.01369	*F*_1,9_ = 8.98	.50	.015
California gull	0.86379	0.04451	*F*_1,160_ = 298.63	.65	<.001
California least tern	0.83616	0.02009	*F*_1,245_ = 189.26	.44	<.001
Caspian tern	0.36835	0.18224	*F*_1,58_ = 7.05	.11	.010
Double‐crested cormorant	0.73191	0.08782	*F*_1,85_ = 63.74	.43	<.001
Forster's tern	0.74602	0.04220	*F*_1,927_ = 349.22	.27	<.001
Western snowy plover	0.73016	0.04372	*F*_1,24_ = 28.32	.54	<.001
Wood duck	0.89982	0.01872	*F*_1,36_ = 66.24	.65	<.001

#### 3.2.4 Relationship between sharp pole and equator eggshell thickness among species

When mean values were used for each species, eggshell thickness at the sharp pole was strongly related to eggshell thickness at the equator (*R* ^2^ = .99; Figure 3b). The slope of the relationship was 0.95, indicating that eggshell thickness at the sharp pole was consistently 95% of the thickness at the equator (sharp pole eggshell thickness = eggshell thickness at the equator × 0.95300 + 0.00029). The 95% CI of the residuals for all species except mallard (*Anas platyrhynchos*), where only 2 eggshells were measured, included zero, indicating that there were no species where the mean relationship between eggshell thickness at the sharp pole and the equator differed from expected.

### 3.3 Eggshell thickness versus egg morphometrics and bird body mass

The relationship between eggshell thickness at the equator and egg length, egg width, or egg volume was stronger among species than within species. Among species, mean species body mass was the best predictor of species mean eggshell thickness, better than species mean egg length, egg width, or egg volume.

#### 3.3.1 Individual species comparisons

**FIGURE 4:**
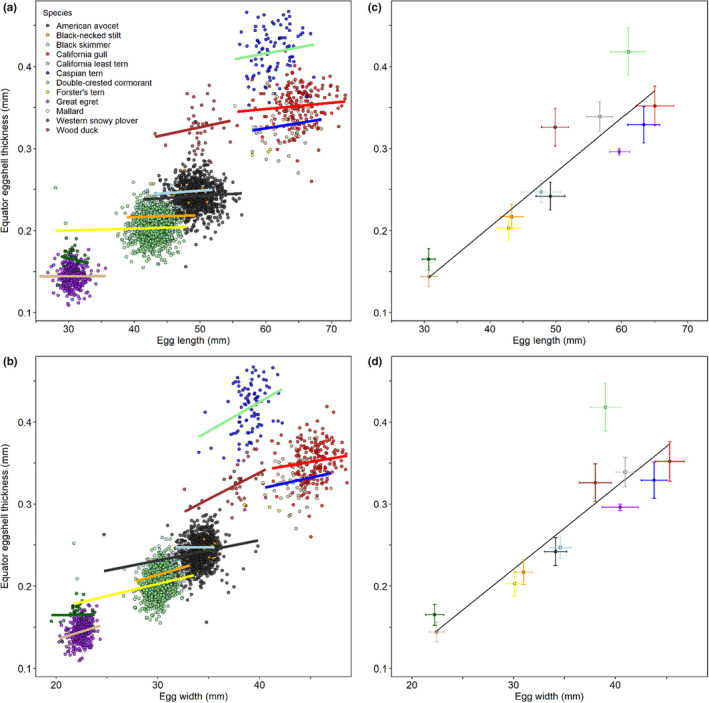
Eggshell thickness at the egg equator as a function of egg length and egg width within and among 12 avian species during 2014–2018. (a) Individual eggs’ equator eggshell thicknesses as a function of egg length within each species (slopes for each species from individual species regressions). (b) Individual eggs’ equator eggshell thicknesses as a function of egg width within each species (slopes for each species from individual species regressions). (c) Arithmetic mean (± *SD*) eggshell thicknesses and egg lengths (raw data) are shown for each species on top of the among‐species regression (equator eggshell thickness [mm] = (0.00664 × egg length [mm]) – 0.06082) based on the species’ mean values. (d) Arithmetic mean (± *SD*) eggshell thicknesses and egg widths (raw data) are shown for each species on top of the among‐species regression (equator eggshell thickness [mm] = (0.00999 × egg width [mm]) – 0.07884) based on the species’ mean values

The slope of the relationship between eggshell thickness and egg morphometrics within each species did not differ among species for length (*F*_11,2844_ = 0.67, *p* = .77; Figure 4) but varied among individual species for width (*F*_11,2844_ = 1.84, *p* = .042; Figure 4) and for volume (*F*_11,2720_ = 2.93, *p* < .001). Within each species, eggshell thickness was not related to egg length for any species (all *F* ≤ 3.50, all *p* ≥ .06; slope coefficients from −0.00198 to 0.00172), but eggshell thickness was related to egg width in 6 species (all *F* ≥ 8.54*, p* ≤ .005, slope coefficients from 0.00247 to 0.00713) but not the remaining 4 species (all *F* ≤ 2.83, *p* ≥ .09; Table 4). Within each of these six species (American avocet, black‐necked stilt, California least tern, double‐crested cormorant, Forster's tern, and wood duck), a 10 mm increase in egg width predicted an increase in eggshell thickness at the equator from 0.025 to 0.071 mm. Similarly, within each species, eggshell thickness was related to egg volume in American avocet, black‐necked stilt, California least tern, double‐crested cormorant, Forster's tern, and wood duck (all *F* ≥ 6.15*, p* ≤ .018) but not in the remaining 4 species (all *F* ≤ 3.31, *p* ≥ .07).

**TABLE 4:** Slope and intercept values for individual species regressions between equator eggshell thickness (mm) and egg length (mm) or egg width (mm) for 10 individual species

Species	Egg length					Egg width				
	Slope	Intercept	*F_df_*	*R* ^2^	*p*	Slope	Intercept	*F_df_*	*R* ^2^	*p*
American avocet	0.0005094	0.21660	*F*_1,840_ = 3.50	<.01	.06	0.00247	0.15728	*F*_1,840_ = 21.65	.03	<.001
Black‐necked stilt	0.0002068	0.20836	*F*_1,199_ = 0.11	<.01	.74	0.00362	0.10513	*F*_1,199_ = 8.54	.04	.004
Black skimmer	0.0005810	0.21971	*F*_1,9_ = 0.17	.02	.69	−0.00004	0.24889	*F*_1,9_ < 0.01	<.01	.99
California gull	0.0007571	0.30291	*F*_1,173_ = 1.47	<.01	.23	0.00210	0.25683	*F*_1,173_ = 2.83	.02	.09
California least tern	0.0000569	0.14267	*F*_1,328_ = 0.01	<.01	.92	0.00382	0.05854	*F*_1,328_ = 15.45	.04	<.001
Caspian tern	0.0012902	0.24736	*F*_1,60_ = 1.21	.02	.28	0.00270	0.21086	*F*_1,60_ = 1.44	.02	.23
Double‐crested cormorant	0.0014791	0.32730	*F*_1,86_ = 1.53	.02	.22	0.00713	0.13931	*F*_1,86_ = 12.24	.12	<.001
Forster's tern	0.0002329	0.19323	*F*_1,1,079_ = 0.84	<.01	.36	0.00296	0.11402	*F*_1,1,079_ = 34.26	.03	<.001
Western snowy plover	−0.0019776	0.22567	*F*_1,32_ = 0.72	.02	.40	0.00006	0.16376	*F*_1,32_ < 0.01	<.01	.98
Wood duck	0.0017174	0.24015	*F*_1,37_ = 0.87	.02	.36	0.00648	0.07923	*F*_1,37_ = 8.94	.19	.005

#### 3.3.2 Among species

Among species, the mean eggshell thickness at the equator increased with mean egg length (*R* ^2^ = .85; Figure 4), mean egg width (*R* ^2^ = .80; Figure 4), and mean egg volume (*R* ^2^ = .79). Across species, an increase in mean egg length of 10 mm predicted an increase in eggshell thickness at the equator of 0.066 mm (eggshell thickness = 0.00664 × egg length–0.06082). Mean egg length for each of the 12 species ranged from approximately 30 mm to 65 mm (Table 2), resulting in a predicted difference of 0.232 mm in the eggshell thickness between the shortest and longest species’ eggs represented by this study. Mean egg width for the 12 species ranged from approximately 22 mm to 45 mm, resulting in a predicted eggshell thickness increase of 0.230 mm between the narrowest and widest species’ eggs in this study (eggshell thickness = 0.00999 × egg width–0.07884; Figure 4).

The average eggshell thickness for most species fell within the expected range for eggshell thickness at the equator, predicted from egg length or egg width, with zero included in the 95% CI of the residuals for all individual eggs from all species except double‐crested cormorant, wood duck, and great egret (*Ardea alba*). Double‐crested cormorant had thicker eggshells at the equator than would be predicted based on the length or width of the egg, and wood duck eggshells also were thicker at the equator than would be predicted based on the length, but not the width, of the egg. Great egret had thinner eggshells than would be predicted based on the length, but not the width, of the egg. The estimated species mean eggshell thickness, using the equation with species mean egg length, ranged from 17.7% lower (double‐crested cormorant) to 13.2% higher (great egret) than the mean measured eggshell thickness. Wood duck (16.9% lower), snowy plover (15.4% lower), Forster's tern (10.3% higher), American avocet (9.5% higher), Caspian tern (9.4% higher), and mallard (6.8% lower) had a predicted mean eggshell thickness that was more than 6.0% higher or lower than the mean measured eggshell thickness. The remaining four species had a predicted mean eggshell thickness that was within 6% of the mean measured eggshell thickness.

The predictive equations using egg morphometrics did not perform well for individual eggs; 47.4% of individual eggs had eggshell thicknesses predicted from egg length that were more than 10% thicker or thinner than the observed eggshell thickness (range 27.3% to 88.6% of eggs within individual species, excluding great egret where the three eggshells were >10% thinner than the predicted eggshell thickness). Similarly, 43.3% of individual eggs had eggshell thicknesses predicted from egg width that were more than 10% thicker or thinner than the observed eggshell thickness (range 25.8% to 100.0% of eggs within individual species, excluding mallard where the 2 eggshells were within 10% of predicted eggshell thickness).

Among species, an increase in log_10_ body mass of 10% predicted an increase in eggshell thickness at the equator of 0.016 mm (*R* ^2^ = .92; *p* < .001). Mean bird mass for the 12 species ranged from approximately 42 g to 1,831 g, resulting in a predicted eggshell thickness increase of 0.261 mm between the birds with the smallest and largest mean body mass in this study (eggshell thickness [mm] = 0.15918 × log_10_(bird body mass [g])–0.12689). The estimated species mean eggshell thicknesses, using this equation, ranged from 20.6% lower (western snowy plover) to 15.5% higher (great egret) than the mean measured eggshell thicknesses. American avocet (14.0% higher), California gull (10.5% lower), California least tern (6.3% lower), and double‐crested cormorant (6.2% higher) had a predicted mean eggshell thickness that was more than 6.0% higher or lower than the mean measured eggshell thickness. The remaining six species had a predicted mean eggshell thickness that was within 6% of the mean measured eggshell thickness.

We compared the four different models to predict species mean eggshell thickness from species mean egg morphometric measurements (egg length, width, or volume) or bird body mass, and log_10_(bird body mass) was the best predictor. The AIC_c_ value of −42.1 for log_10_(bird body mass) was more than a ΔAIC_c_ of 2 from the models using egg length (ΔAIC_c_ = 5.0), egg width (ΔAIC_c_ = 8.3), or egg volume (ΔAIC_c_ = 8.8).

### 3.4 Eggshell thickness versus embryo age

We did not find support for a decrease in the eggshell thickness (including the eggshell membrane) with increasing embryonic development for a subset of normally developing American avocet, black‐necked stilt, and Forster's tern eggs (mean embryo age 7.0 ± 3.9 days; interquartile range 4–10 days; range 0–23 days), after removing the nonsignificant species × embryo age interaction term (*F*_2,1198_ = 1.52, *p* = .22). Instead, we observed a positive, although biologically small, increase in eggshell thickness at the equator with embryonic age (*F*_1,1200_ = 5.85, *p* = .016), after accounting for differences among species (*F*_2,1200_ = 980.87, *p* < .001). However, the variability of eggshell thickness at the equator within a species was far greater than any effect of embryonic age. For example, after excluding the 5% thinnest and the 5% thickest eggshell measurements, eggshell thickness measurements at the equator ranged 0.055 mm for American avocet, 0.050 mm for black‐necked stilt, and 0.049 mm for Forster's tern. In contrast, the eggshell thickness at the equator increased by 0.0066 mm during a standard 24‐day incubation period (mean incubation duration is 22 days for American avocets, 23 days for black‐necked stilt, and 24 days for Forster's tern), which is approximately 3.3% of the average equator eggshell thickness for Forster's tern. For American avocet, 0.0066 mm is 2.7% of the average equator eggshell thickness and it is 3.0% of the average equator eggshell thickness for black‐necked stilt. For the model assessing eggshell thickness at the sharp pole, we did not detect any change in eggshell thickness at the sharp pole with embryonic age (*F*_1,1160_ = 0.09, *p* = .77) when we accounted for differences among species (*F*_2,1160_ = 318.57, *p* < .001), after removing the nonsignificant species × embryo age interaction term (*F*_2,1158_ = 0.26, *p* = .77).

For embryos older than 7 days (mean 10.6 ± 2.5 days, interquartile range: 9–12 days; range: 8–23 days), after removing the nonsignificant species × embryo age interaction term (*F*_2,513_ = 0.28, *p* = .76), we did not find support for any change in eggshell thickness (including the eggshell membrane) at the equator with increasing embryonic development (*F*_1,515_ = 0.03, *p* = .86), after accounting for differences among species (*F*_2,515_ = 419.15, *p* < .001). Of note, only 3.5% of eggs had embryos that were in the final quarter of embryonic development. For the sharp pole in eggshells with embryos older than 7 days, after removing the nonsignificant species × embryo age interaction term (*F*_2,490_ = 0.21, *p* = .81), we did not find support for any change in eggshell thickness (including the eggshell membrane) at the equator with increasing embryonic development (*F*_1,492_ = 0.32, *p* = .57), after accounting for differences among species (*F*_2,492_ = 146.31, *p* < .001).

### 3.5 Eggshell thickness versus egg status

**FIGURE 5:**
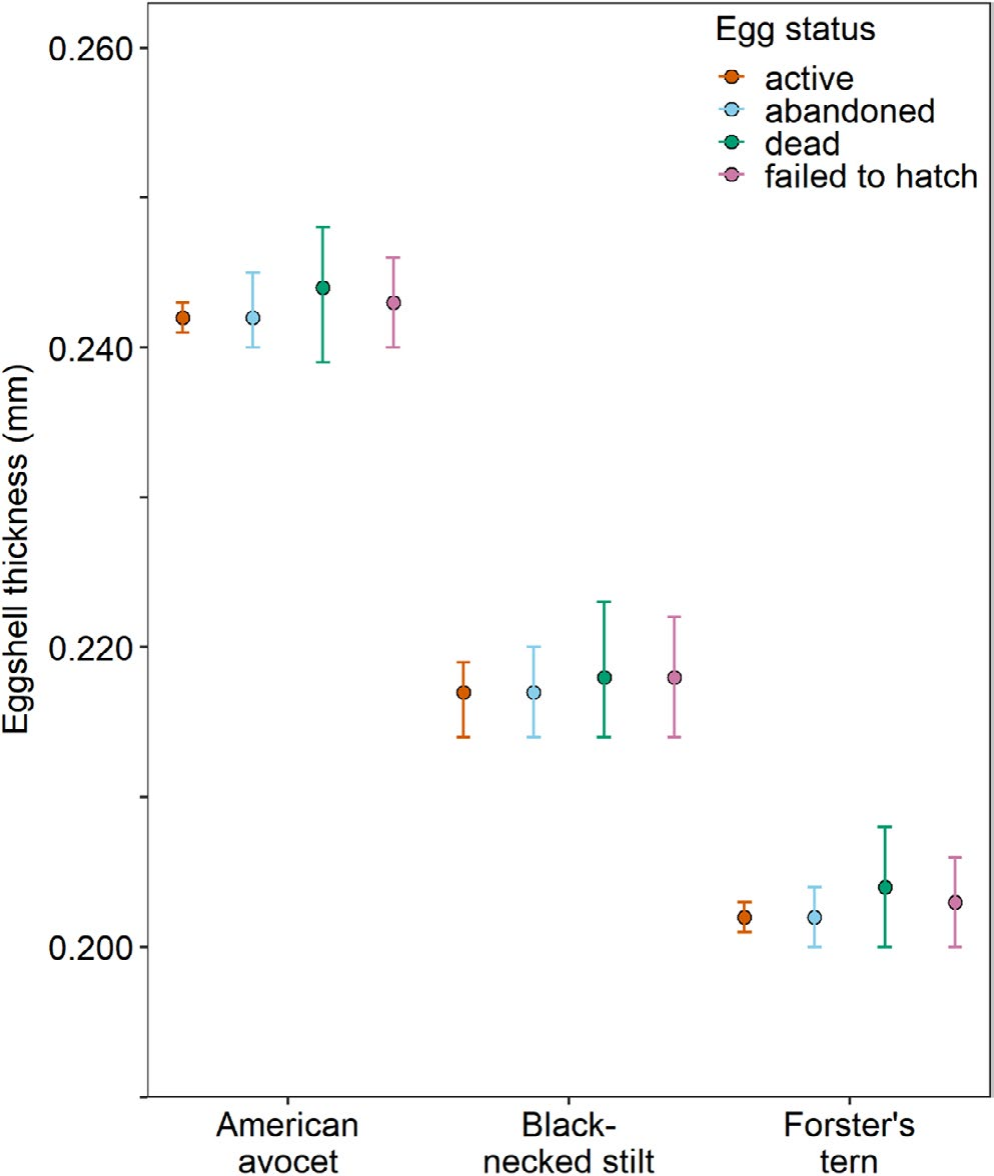
Eggshell thickness at the egg equator (model‐generated least squares mean ± 95% CI) did not relate to the status of the egg at the time of collection for three avian species (American avocet [*Recurvirostra americana*], black‐necked stilt [*Himantopus mexicanus*], and Forster's tern [*Sterna forsteri*]) during 2014–2018. Active eggs were normally progressing in nests that were actively being incubated, whereas abandoned eggs were from nests where the parents had naturally abandoned the nest. Eggs classified as dead contained dead embryos and had stopped progressing normally in nests while they were still being incubated and no sibling eggs in the clutch hatched. Failed‐to‐hatch eggs contained dead embryos and also did not hatch but were from nests where other sibling eggs in the clutch successfully hatched

For a subset of American avocet, black‐necked stilt, and Forster's tern eggs that were sampled during weekly nest monitoring visits, the status of each egg was categorized upon collection as active, abandoned, dead, or failed to hatch. Embryo ages were 7.0 ± 3.9 days (interquartile range 4–10 days) for eggs from active nests (active egg status), 8.8 ± 6.0 days (interquartile range 3–14 days) for eggs that were naturally abandoned by the parents (abandoned egg status), 12.1 ± 7.2 days (interquartile range 3–18 days) for eggs with dead embryos where no sibling eggs hatched from the nest (dead egg status), and 11.6 ± 6.9 days (interquartile range 5–18 days) for eggs with dead embryos where sibling eggs hatched from the nest (failed‐to‐hatch egg status). At the egg equator, after removing the nonsignificant egg status × species interaction term (*F*_6,1015.0_ = 1.64, *p* = .13), eggshell thickness did not differ among egg status (*F*_3,1281.4_ = 0.32, *p* = .81; Figure 5) after accounting for species (*F*_2,1438.8_ = 1,089.71, *p* < .001) and embryo age (*F*_1,1497.4_ = 5.05, *p* = .02). Similarly, at the sharp pole, after removing the nonsignificant egg status × species interaction term (*F*_6,990.0_ = 0.84, *p* = .54) and accounting for species (*F*_2,1340.1_ = 1,047.1, *p* < .001) and embryo age (*F*_1,1394.6_ = 2.45, *p* = .12), there was no detectable effect of egg status (*F*_3,1141.0_ = 0.68, *p* = .57) on eggshell thickness.

### 3.6 Eggshell thickness versus egg content THg

**FIGURE 6:**
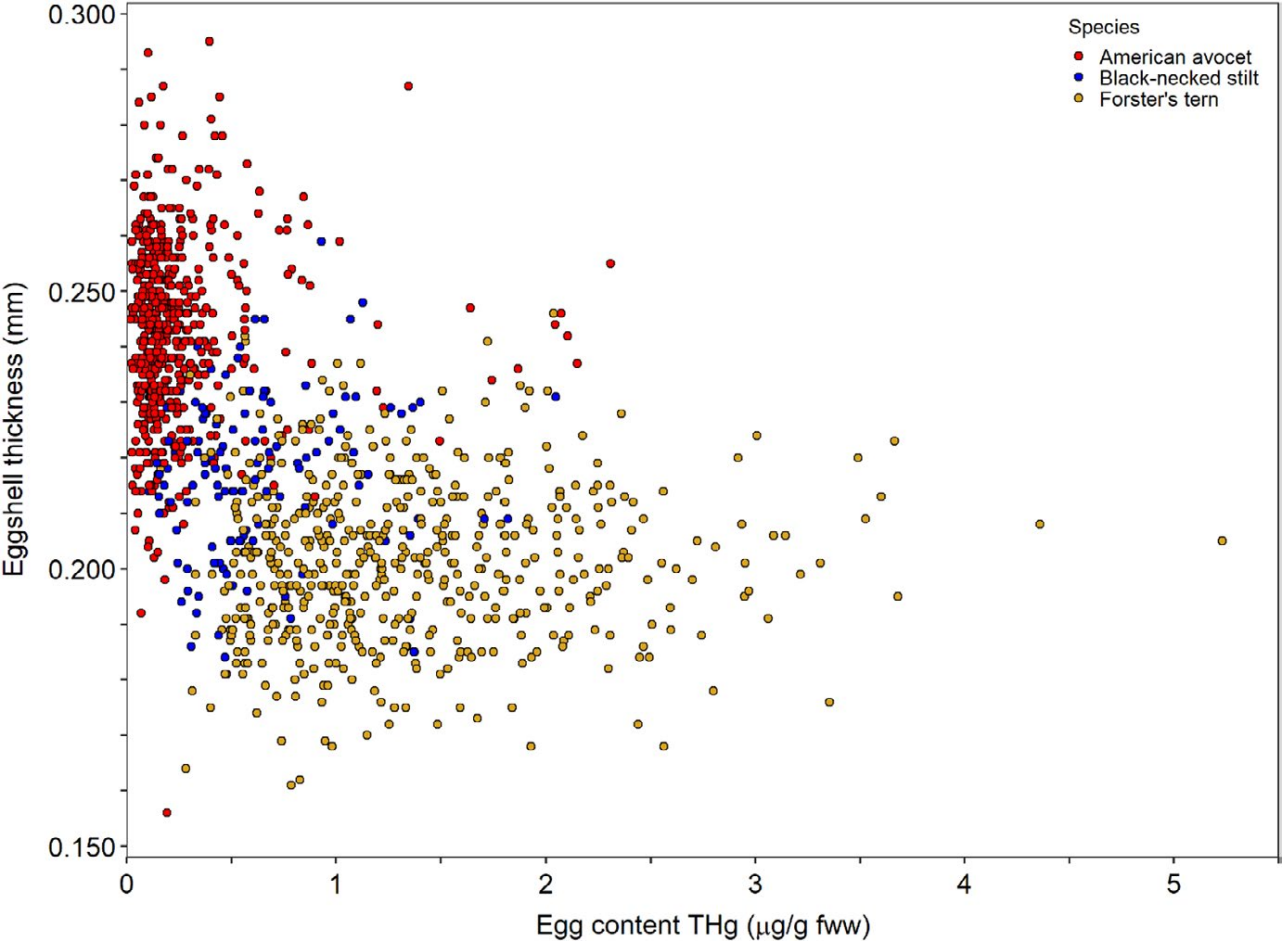
Eggshell thickness at the egg equator was not related to total mercury (THg) concentrations in the egg contents, quantified using fresh wet weight (fww), for three avian species (American avocet [*Recurvirostra americana*], black‐necked stilt [*Himantopus mexicanus*], and Forster's tern [*Sterna forsteri*]) during 2014–2018

We did not detect a relationship between eggshell thickness and egg content THg concentrations. At the equator, after removing the nonsignificant egg content THg concentration × species interaction term (*F*_2,1187_ = 0.36, *p* = .70), eggshell thickness was not related to the egg content THg concentration (fww) (*F*_1,1189_ = 1.67, *p* = .20; Figure 6), after accounting for species (*F*_2,1189_ = 532.24, *p* < .001) and embryo age (*F*_1,1189_ = 5.39, *p* = .02). Similarly, at the sharp pole, after removing the nonsignificant egg content THg concentration × species interaction term (*F*_2,1138_ = 0.05, *p* = .95), eggshell thickness was not related to the egg content THg concentration (fww) (*F*_1,1140_ = 0.12, *p* = .73), after accounting for species (*F*_2,1140_ = 164.60, *p* < .001) and embryo age (*F*_1,1140_ = 0.07, *p* = .79).

## 4 DISCUSSION

We present eggshell thickness measurements for an additional two avian species in North America (California gull and mallard) that have not been previously measured with the eggshell membrane attached, and we compare eggshell thickness for another 10 species to previous literature (Table 5). Many of the previously measured species were initially sampled to test for effects of organochlorine contaminants on eggshell thickness (Anderson & Hickey, 1972; Blus & Prouty, 1979; Burger, Viscido, & Gochfeld, 1995; Gress, Risebrough, Anderson, Kiff, & Jehl, 1973; Henny, Blus, & Prouty, 1982; King, Flickinger, & Hildebrand, 1978; Postupalsky, 1997; Roberts, 1997). For the 10 species that had been previously measured, the mean eggshell thicknesses were similar to previous studies in North America with some slight differences among studies (Table 5). For example, mean American avocet and black‐necked stilt eggshells were 10.0% and 3.8% thicker, respectively, in the present study than previous mean eggshell thickness measurements for the same species in Utah (Henny, Anderson, & Crayon, 2008), although the ranges of eggshell thicknesses observed in the two studies were similar. Black skimmer eggshells were, on average, 5.6% to 10.3% thicker in the present study than in southern California (Roberts, 1997; Santolo, 2018), but were ≥42.3% thinner than the mean of the most recently sampled eggshells (1990s) of black skimmer from the east coast of the United States (Burger et al., 1995). Forster's tern eggshell thickness in the present study was similar to Forster's tern eggshell thickness from other regions in California (Grant, 1982; Roberts, 1997; Santolo, 2018). However, comparisons of eggshell thickness measurements with previous studies are complicated by the potential influence of geography and subspecies on eggshell thickness in addition to local contamination by DDT and other chlorinated pesticides that might decrease eggshell thickness (Anderson & Hickey, 1972). Many previous studies that examined eggshell thickness in birds were conducted specifically to look for eggshell thinning as a result of environmental contamination. Furthermore, our methodology of using newer technology (a Hall effect thickness gauge) provides a more precise and repeatable thickness measurement than analog micrometers that have been used in many previous studies (Santolo, 2018). Additionally, the Hall effect thickness gauge reduces the potential for the eggshell membrane to be compressed while obtaining the eggshell thickness measurement.

**TABLE 5:** Eggshell thickness measurements (mm) from the present study (raw data) and the literature

Species	Collection location	Collection year	Sample size	Mean eggshell thickness (mm) ± *SD*, *SE* or CL (range)	Position of measurement on egg	Eggshell membrane included in measurement (yes/no/not specified)	Measurement method	Reference
American avocet	California	1895–1936	19	0.249 ± 0.006*^SD^*	Equator	Yes	Magna‐Mike 8600 HETG	Santolo et al. (2016)
	Salton Sea, California	1975–1978	7	0.236 ± 0.005*^SD^*	Not specified	Yes	Micrometer	Grant (1982)
	Nevada	1991	32	0.262 ± 0.022 (0.220–0.310)	Not specified	Yes	Screw‐type caliper	Ackerman, Hartman, et al. (2013)
	Utah	2004	3	0.220 ± 0.027*^SD^*	Equator	Yes	Modified Starrett micrometer	Henny et al. (2008)
	Newport Bay, California	2004–2005	13	0.243 ± 0.020*^SD^*	Equator	Yes	Modified Starrett micrometer	Santolo et al. (2016)
	Great Salt Lake, Utah	2010	6	0.228 ± 0.019*^SD^*	Not specified	Yes	Starrett micrometer	Cavitt, Linford, and Wilson (2010)
	Great Salt Lake, Utah	2011	10	0.254 ± 0.021*^SD^*	Not specified	Yes	Starrett micrometer	Cavitt and Wilson (2011)
	Newport Bay, California	2013–2016	37	0.237	Equator	Yes	Magna‐Mike 8600 HETG	Santolo (2018)
	Newport Bay, California*	2013–2016	37	0.231	Equator	Yes	Modified Starrett micrometer	Santolo (2018)
	California	2014–2018	843	0.242 ± 0.017*^SD^* (0.156–0.297)	Equator	Yes	Magna‐Mike 8600 HETG	Present study
Black‐necked stilt	Utah	Pre‐DDT^a^	40	0.205 ± 0.013*^SE^*	Not specified	Not specified	Micrometer	Henny, Blus, and Hulse (1985)
	California	1893–1930	56	0.214 ± 0.012*^SD^*	Equator	Yes	Magna‐Mike 8600 HETG	Santolo et al. (2016)
	Salton Sea, California	1975–1978	22	0.224 ± 0.017*^SD^*	Not specified	Yes	Micrometer	Grant (1982)
	Carson Lake, Nevada	1980	10	0.217^geo^	Equator	Not specified	Micrometer	Henny et al. (1985)
	Carson Lake, Nevada	1981	10	0.203^geo^	Equator	Not specified	Micrometer	Henny et al. (1985)
	Carson Lake, Nevada	1982	10	0.209^geo^	Equator	Not specified	Micrometer	Henny et al. (1985)
	Carson Lake, Nevada	1983	10	0.217^geo^	Equator	Not specified	Micrometer	Henny et al. (1985)
	California	1985–2007	5	0.236 ± 0.015*^SD^*	Equator	Yes	Starrett micrometer	Mora, Brattin, Baxter, and Rivers (2011)
	Nevada	1991	18	0.234 ± 0.022 (0.210–0.280)	Not specified	Yes	Screw‐type caliper	Robinson et al. (1999)
	Utah	2004	12	0.209 ± 0.011*^SD^*	Equator	Yes	Modified Starrett micrometer	Henny et al. (2008)
	Newport Bay, California	2004–2005	13	0.214 ± 0.017*^SD^*	Equator	Yes	Modified Starrett micrometer	Santolo et al. (2016)
	Great Salt Lake, Utah	2010	5	0.210 ± 0.027*^SD^*	Not specified	Yes	Starrett micrometer	Cavitt et al. (2010)
	Newport Bay, California	2013–2016	36	0.208	Equator	Yes	Magna‐Mike 8600 HETG	Santolo (2018)
	Newport Bay, California*	2013–2016	36	0.199	Equator	Yes	Modified Starrett micrometer	Santolo (2018)
	San Francisco Bay, California	2014–2018	201	0.217 ± 0.015*^SD^* (0.184–0.259)	Equator	Yes	Magna‐Mike 8600 HETG	Present study
Black skimmer	Texas^b^	Pre‐1931^c^	28	0.24 ± 0.004*^SE^*	Not specified	Yes	Micrometer	White, Mitchell, and Swineford (1984)
	Texas	Pre‐1943^c^	28	0.249 ± 0.004*^SE^*	Not specified	Yes	Micrometer	King et al. (1978)
	South Carolina	Pre‐1947	241	0.229 ± 0.001*^SE^*	Not specified	Yes	Micrometer	Blus and Stafford (1980)
	South Carolina	1969	10	0.217 ± 0.004*^SE^*	Equator	Yes	Micrometer	Blus and Stafford (1980)
	Galveston Bay, Texas	1970	48	0.244 ± 0.002*^SE^* (0.22–0.28)	Equator	Yes	Micrometer	King and Krynitsky (1986)
	New York	1970s	31	0.366 ± 0.010*^SE^*	Equator	Yes	Micrometer	Burger et al. (1995)
	South Carolina	1971	26	0.238 ± 0.004*^SE^*	Equator	Yes	Micrometer	Blus and Stafford (1980)
	South Carolina	1972	11	0.218 ± 0.004*^SE^*	Equator	Yes	Micrometer	Blus and Stafford (1980)
	South Carolina	1973	21	0.224 ± 0.004*^SE^*	Equator	Yes	Micrometer	Blus and Stafford (1980)
	South Carolina	1974	12	0.227 ± 0.005*^SE^*	Equator	Yes	Micrometer	Blus and Stafford (1980)
	South Carolina	1975	23	0.221 ± 0.003*^SE^*	Equator	Yes	Micrometer	Blus and Stafford (1980)
	Corpus Christi, Texas	1978	12	0.21 ± 0.003*^SE^*	Equator	Yes	Micrometer	White et al. (1984)
	Corpus Christi, Texas	1979	40	0.22 ± 0.002*^SE^*	Equator	Yes	Micrometer	White et al. (1984)
	Port Mansfield, Texas	1979	24	0.23 ± 0.003*^SE^*	Equator	Yes	Micrometer	White et al. (1984)
	Laguna Vista, Texas	1979	22	0.23 ± 0.003*^SE^*	Equator	Yes	Micrometer	White et al. (1984)
	Corpus Christi, Texas	1980	19	0.24 ± 0.003*^SE^*	Equator	Yes	Micrometer	White et al. (1984)
	Port Mansfield, Texas	1980	21	0.23 ± 0.003*^SE^*	Equator	Yes	Micrometer	White et al. (1984)
	Laguna Vista, Texas	1980	20	0.22 ± 0.003*^SE^*	Equator	Yes	Micrometer	White et al. (1984)
	Galveston Bay, Texas	1980	57	0.235 ± 0.003*^SE^* (0.21–0.28)	Equator	Yes	Micrometer	King and Krynitsky (1986)
	New York	1980s	45	0.363 ± 0.005*^SE^*	Equator	Yes	Micrometer	Burger et al. (1995)
	New Jersey	1980s	16	0.351 ± 0.010*^SE^*	Equator	Yes	Micrometer	Burger et al. (1995)
	Galveston Bay, Texas	1981	41	0.245 ± 0.002*^SE^* (0.20–0.29)	Equator	Yes	Micrometer	King and Krynitsky (1986)
	Corpus Christi, Texas	1981	15	0.23 ± 0.004*^SE^*	Equator	Yes	Micrometer	White et al. (1984)
	Port Mansfield, Texas	1981	13	0.22 ± 0.003*^SE^*	Equator	Yes	Micrometer	White et al. (1984)
	Laguna Vista, Texas	1981	15	0.22 ± 0.005*^SE^*	Equator	Yes	Micrometer	White et al. (1984)
	Galveston Bay, Texas	1982	48	0.238 ± 0.002*^SE^* (0.21–0.26)	Equator	Yes	Micrometer	King and Krynitsky (1986)
	Texas	1984	≥41	0.240	Equator	Yes	Micrometer	King et al. (1991)
	Laguna Vista, Texas	1984	80	0.24 ± 0.01*^SD^*	Equator	Yes	Micrometer	Custer and Mitchell (1987)
	Not specified	Pre‐1985	5	0.200	Not specified	No	Ball‐point caliper	Ar and Rahn (1985)
	New York	1990s	49	0.546 ± 0.005*^SE^*	Equator	Yes	Micrometer	Burger et al. (1995)
	New Jersey	1990s	13	0.428 ± 0.008*^SE^*	Equator	Yes	Micrometer	Burger et al. (1995)
	San Diego Bay, California	1991	6	0.234 (0.215–0.253)	Equator	Yes	Modified Mitutoyo Micrometer	Roberts (1997)
	San Diego Bay, California	1993–1994	22	0.229 (0.211–0.258)	Equator	Yes	Modified Mitutoyo micrometer	Roberts (1997)
	Newport Bay, California	2013–2016	11	0.224	Equator	Yes	Magna‐Mike 8600 HETG	Santolo (2018)
	Newport Bay, California*	2013–2016	11	0.224	Equator	Yes	Modified Starrett micrometer	Santolo (2018)
	San Francisco Bay, California	2014–2018	11	0.247 ± 0.013 (0.229–0.273)*^SD^*	Equator	Yes	Magna‐Mike 8600 HETG	Present study
California gull	San Francisco Bay, California	2014–2018	175	0.352 ± 0.024 (0.260–0.419)*^SD^*	Equator	Yes	Magna‐Mike 8600 HETG	Present study
Caspian tern	Pacific Northwest	1911–1931	8	0.34 ± 0.03	Not specified	Yes	Not specified	Penland (1981)
	Not specified	1941–1945	5	0.346 ± 0.015*^SD^*	Equator	Yes	Modified Starrett micrometer	Ohlendorf, Schaffner, Custer, Stafford, and Charles (1985)
	Texas	Pre‐1943	15	0.336 ± 0.005*^SE^*	Not specified	Yes	Micrometer	King et al. (1978)
	San Francisco Bay, California	Pre‐1945	44	0.329 ± 0.017*^SD^*	Equator	Yes	Modified Starrett micrometer	Parkin (1998)
	Not specified	1960–1964	5	0.320 ± 0.010*^SD^*	Equator	Yes	Modified Starrett micrometer	Ohlendorf et al. (1985)
	Grays Harbor, Washington	1961–1976	12	0.35 ± 0.03	Not specified	Yes	Not specified	Penland (1981)
	Texas	1970	32	0.337 ± 0.003*^SE^*	Equator	Yes	Micrometer	King et al. (1978)
	San Francisco Bay, California	1971	6	0.323 ± 0.013*^SD^*	Equator	Yes	Modified Starrett micrometer	Parkin (1998)
	San Francisco Bay, California	1971	6	0.329 ± 0.012*^SD^*	Equator	Yes	Modified Starrett micrometer	Parkin (1998)
	Bothnian Bay, Finland	1977–1978	118	0.225 ± 0.001*^SE^* (0.150–0.258)	Equator	No	Binocular microscope	Pulliainen and Marjakangas (1980)
	Lake Michigan, Michigan	1980	11	0.324 ± 0.02*^SD^*	Not specified	Not specified	Dial micrometer with ball attachment	Struger and Weseloh (1985)
	Lake Michigan, Michigan	1980	10	0.338 ± 0.02*^SD^*	Not specified	Not specified	Dial micrometer with ball attachment	Struger and Weseloh (1985)
	Lake Michigan, Michigan	1980	10	0.338 ± 0.03*^SD^*	Not specified	Not specified	dial micrometer with ball attachment	Struger and Weseloh (1985)
	Lake Huron, Ontario, Canada	1980	10	0.314 ± 0.02*^SD^*	Not specified	Not specified	Dial micrometer with ball attachment	Struger and Weseloh (1985)
	Lake Huron, Ontario, Canada	1980	10	0.303 ± 0.02*^SD^*	Not specified	Not specified	Dial micrometer with ball attachment	Struger and Weseloh (1985)
	Lake Huron, Ontario, Canada	1980	9	0.318 ± 0.02*^SD^*	Not specified	Not specified	Dial micrometer with ball attachment	Struger and Weseloh (1985)
	Lake Ontario, Ontario, Canada	1981	10	0.329 ± 0.02*^SD^*	Not specified	Not specified	Dial micrometer with ball attachment	Struger and Weseloh (1985)
	San Diego Bay, California	1981	25	0.33	Equator	Yes	Modified Starrett Micrometer	Ohlendorf et al. (1985)
	San Diego Bay, California	1991	1	0.339	Equator	Yes	Modified Mitutoyo micrometer	Roberts (1997)
	Great Lakes, Ontario, Canada	1991	108	0.332	Equator	Yes	Starrett micrometer	Ewins et al. (1994)
	San Diego Bay, California	1993–1994	9	0.329 (0.275–0.358)	Equator	Yes	Modified Mitutoyo micrometer	Roberts (1997)
	Elkhorn Slough, California	1994	4	0.302 ± 0.004*^SD^*	Equator	Yes	Modified Starrett micrometer	Parkin (1998)
	Elkhorn Slough, California	1995	48	0.294 ± 0.008*^SD^*	Equator	Yes	Modified Starrett micrometer	Parkin (1998)
	Salinas River, California	1996	13	0.290 ± 0.003*^SD^*	Equator	Yes	Modified Starrett micrometer	Parkin (1998)
	California	2013	15	0.341 (0.300–0.371)	Equator	Yes	Starrett electronic digital micrometer	Clatterbuck, Lewison, Dodder, Zeeman, and Schiff (2018)
	San Francisco Bay, California; Washington	2017–2018	62	0.329 ± 0.022*^SD^* (0.270–0.381)	Equator	Yes	Magna‐Mike 8600 HETG	Present study
Double‐crested cormorant^d^								
*P. a. albociliatus*	Pacific Northwest	pre−1947	51	0.432 ± 0.005*^SE^*	Not specified	Yes	Micrometer	Henny et al. (1982)
	California; Mexico	pre−1947	29	0.428 ± 0.012^CL^	Equator	Yes	Micrometer	Anderson and Hickey (1972)
	Pacific Northwest	1949–1953	14	0.386 ± 0.011*^SE^*	Equator	Yes	Micrometer	Henny et al. (1982)
	Pacific Northwest	1954–1958	11	0.374 ± 0.007*^SE^*	Equator	Yes	Micrometer	Henny et al. (1982)
	Anacapa, California	1969	7	0.38 ± 0.02^CL^	Equator	Yes	Micrometer	Gress et al. (1973)
	Los Coronados, Mexico	1969	6	0.30 ± 0.03^CL^	Equator	Yes	Micrometer	Gress et al. (1973)
	San Martin, Mexico	1969	7	0.44 ± 0.02^CL^	Equator	Yes	Micrometer	Gress et al. (1973)
	Klamath NWR, Oregon	1977	5	0.413 ± 0.033*^SD^*	Equator	Yes	Micrometer	Henny, Blus, Thompson, and Wilson (1989)
	Oregon	1979	10	0.432 ± 0.045*^SD^*	Equator	Yes	Micrometer	Henny et al. (1982)
	Malheur NWR, Oregon	1980	6	0.442 ± 0.024*^SD^*	Equator	Yes	Micrometer	Henny et al. (1989)
	Grays Harbor, Washington	1984	14	0.453 ± 0.063*^SD^*	Equator	Yes	Modified bench comparator	Speich et al. (1992)
	Puget Sound, Washington	1984	11	0.454 ± 0.039*^SD^*	Equator	Yes	Micrometer	Henny et al. (1989)
	Puget Sound, Washington	1984	36	0.408 ± 0.033*^SD^*	Equator	Yes	Micrometer	Henny et al. (1989)
	Columbia River, Washington	1990–1995	31	0.428 ± 0.040*^SD^* (0.370–0.530)	Equator	Yes	Dial micrometer	Buck and Sproul (1999)
	Columbia River, Washington	1990–1995	37	0.416 ± 0.042*^SD^* (0.297–0.479)	Equator	Yes	Dial micrometer	Buck and Sproul (1999)
	Oregon Coast, Oregon	1992	15	0.428 ± 0.040*^SD^* (0.370–0.530)	Not specified	Not specified	Not specified	Buck and Sproul (1999)
	California	2013	8	0.406 (0.328–0.466)	Equator	Yes	Starrett electronic digital micrometer	Clatterbuck et al. (2018)
	California	2016–2018	88	0.433 ± 0.033*^SD^*	Equator	Yes	Magna‐Mike 8600 HETG	Present study
*P. a. auritus*	Interior North America	Pre‐1947	350	0.430 ± 0.003^CL^	Equator	Yes	Micrometer	Anderson and Hickey (1972)
	Ontario	1947	6	0.440 ± 0.013^CL^	Equator	Yes	Micrometer	Anderson and Hickey (1972)
	Manitoba, Canada	1952	11	0.406 ± 0.015*^SE^*	Equator	Yes	Micrometer	Anderson and Hickey (1972)
	Ontario	1959–1961	7	0.340 ± 0.015^CL^	Equator	Yes	Micrometer	Anderson and Hickey (1972)
	Interior North America	1965–1967	76	0.399 ± 0.014^CL^	Equator	Yes	Micrometer	Anderson and Hickey (1972)
	Wisconsin	1965–1968	5	0.309 ± 0.010^CL^	Equator	Yes	Micrometer	Anderson and Hickey (1972)
	Ontario, Canada	1969	23	0.315 (0.22–0.39)	Equator	Yes	Micrometer	Postupalsky (1997)
	Ontario, Canada	1969	39	0.340 ± 0.020^CL^	Equator	Yes	Micrometer	Anderson and Hickey (1972)
	Ontario, Canada	1970	39	0.317 (0.18–0.41)	Equator	Yes	Micrometer	Postupalsky (1997)
	Ontario, Canada	1970	40	0.272 (0.17–0.37)	Equator	Yes	Micrometer	Postupalsky (1997)
	Ontario, Canada	1971	28	0.352 (0.25–0.44)	Equator	Yes	Micrometer	Postupalsky (1997)
	Ontario, Canada	1971	40	0.303 (0.17–0.40)	Equator	Yes	Micrometer	Postupalsky (1997)
	Ontario, Canada	1972	18	0.327 ± 0.023*^SD^*	Not specified	Not specified	Not specified	Weseloh, Teeple, and Gilbertson (1983)
	Ontario, Canada	1972–1973	9	0.392	Equator	Yes	Micrometer	Postupalsky (1997)
	Lake Michigan, Michigan	1977	4	0.370	Equator	Yes	Not specified	Heinz, Erdman, Haseltine, and Stafford (1985)
	Wisconsin	1977	3	0.373	Equator	Yes	Not specified	Heinz et al. (1985)
	Lake Michigan, Michigan	1978	5	0.397	Equator	Yes	Not specified	Heinz et al. (1985)
	Lake Michigan, Michigan	1978	1	0.427	Equator	Yes	Not specified	Heinz et al. (1985)
	Wisconsin	1980	3	0.423	Equator	Yes	Not specified	Heinz et al. (1985)
	Alberta, Canada	1984–1985	127	0.44 ± 0.04	Equator	Yes	Micrometer	Somers, Goski, and Barbeau (1993)
	Minnesota, Wisconsin	1994–1995	306	0.410 (0.313–0.501)	Equator	Yes	Micrometer	Custer et al. (1999)
Forster's tern	Monterey County, California	1932–1939	60	0.192 (0.166–0.215)	Equator	Yes	Magna‐Mike 8600 HETG	Santolo (2018)
	Texas^b^	pre−1943	26	0.219 ± 0.003*^SE^*	Not specified	Yes	Micrometer	King et al. (1978)
	Texas	1970	41	0.218 ± 0.003*^SE^*	Equator	Yes	Micrometer	King et al. (1978)
	Salton Sea, California	1975–1978	7	0.206 ± 0.014*^SD^*	Not specified	Yes	Micrometer	Grant (1982)
	San Diego Bay, California	1981	3	0.206 ± 0.014*^SD^*	Equator	Yes	Modified Starrett micrometer	Ohlendorf et al. (1985)
	San Diego Bay, California	1991	12	0.201 (0.188–0.216)	Equator	Yes	Modified Mitutoyo micrometer	Roberts (1997)
	San Diego Bay, California	1994	2	0.208 (0.201–0.216)	Equator	Yes	Modified Mitutoyo micrometer	Roberts (1997)
	Newport Bay, California	2013–2016	27	0.198	Equator	Yes	Magna‐Mike 8600 HETG	Santolo (2018)
	San Francisco Bay, California	2014–2018	1,085	0.203 ± 0.015*^SD^* (0.155–0.257)	Equator	Yes	Magna‐Mike 8600 HETG	Present study
Great egret	Florida, South Carolina	Pre‐1943	30	0.295 ± 0.004*^SE^*	Not specified	Yes	Micrometer	King et al. (1978)
	Not specified	Pre‐1947	235	0.295 ± 0.003^CL^	Equator	Yes	Micrometer	Faber, Risebrough, and Pratt (1972)
	California	1969–1970	64	0.250 ± 0.007^CL^	Equator	Yes	Micrometer	Faber et al. (1972)
	California	1969–1970	13	0.272 ± 0.013^CL^	Equator	Yes	Micrometer	Faber et al. (1972)
	California	1969–1970	51	0.244 ± 0.008^CL^	Equator	Yes	Micrometer	Faber et al. (1972)
	Texas	1970	113	0.282 ± 0.002*^SE^*	Equator	Yes	Micrometer	King et al. (1978)
	Salton Sea, California	1985	11	0.244 ± 0.016*^SD^*	Equator	Yes	Modified Starrett micrometer	Ohlendorf and Marois (1990)
	Salton Sea, California	1993	29	0.282 ± 0.024*^SD^*	Not specified	Not specified	Not specified	Bennett (1998)
	Utah	2004	12	0.289 ± 0.012*^SD^*	Equator	Yes	Modified Starrett micrometer	Henny et al. (2008)
	California	2018	3	0.296 ± 0.004*^SD^* (0.292–0.299)	Equator	Yes	Magna‐Mike 8600 HETG	Present study
Least tern^e^	Texas^b^	Pre‐1943	22	0.156 ± 0.003*^SE^*	Not specified	Yes	Micrometer	King et al. (1978)
	South Carolina	Pre‐1947	61	0.152 ± 0.002*^SE^*	Equator	Yes	Micrometer	Blus and Prouty (1979)
	New Jersey	1970s	31	0.366 ± 0.012*^SE^*	Equator	Yes	Micrometer	Burger et al. (1995)
	Texas	1970	15	0.154 ± 0.004*^SE^*	Equator	Yes	Micrometer	King et al. (1978)
	South Carolina	1972	11	0.145 ± 0.005*^SE^*	Equator	Yes	Micrometer	Blus and Prouty (1979)
	Massachusetts	1974	12	0.13 ± 0.01*^SD^*	Equator	Yes	Micrometer	Rahn, Paganelli, Nisbet, and Whittow (1976)
	South Carolina	1974	20	0.142 ± 0.002*^SE^*	Equator	Yes	Micrometer	Blus and Prouty (1979)
	South Carolina	1975	15	0.149 ± 0.004*^SE^*	Equator	Yes	Micrometer	Blus and Prouty (1979)
	New Jersey	1980s	20	0.338 ± 0.013*^SE^*	Equator	Yes	Micrometer	Burger et al. (1995)
	South Dakota	1989–1991	99	0.245 ± 0.030*^SD^*	Not specified	Yes	Micrometer	Ruelle (1993)
	New Jersey	1990s	23	0.410 ± 0.013*^SE^*	Equator	Yes	Micrometer	Burger et al. (1995)
	Kansas	1991–1994	16	0.160 ± 0.005*^SE^*	Equator	Yes	Starrett pocket dial gauge 1010RZ	Koenen and Leslie (1996)
	Oklahoma	1993–1994	80	0.153 ± 0.002*^SE^*	Equator	Yes	Starret pocket dial gauge 1010RZ	Koenen and Leslie (1996)
California least tern (*S. a. browni*)	California	Pre‐1947	32	0.154 ± 0.002*^SE^* (0.13–0.18)	Not specified	Yes	Dial micrometer	Massey (1972)
	California	1970–1971	22	0.148 ± 0.003*^SE^* (0.13–0.18)	Not specified	Yes	Dial micrometer	Massey (1972)
	California	1981–1985	16	0.151 ± 0.001*^SE^*	Not specified	Yes	Federal 35 bench comparator	Boardman (1998)
	San Diego Bay, California	1990–1991	21	0.149 (0.109–0.174)	Equator	Yes	Modified Mitutoyo micrometer	Roberts (1997)
	San Diego Bay, California	1990–1992	28	0.148 (0.134–0.156)	Equator	Yes	Modified Mitutoyo micrometer	Roberts (1997)
	San Diego Bay, California	1990–1992	27	0.144 (0.125–0.166)	Equator	Yes	Modified Mitutoyo micrometer	Roberts (1997)
	San Diego Bay, California	1991	2	0.152 (0.140–0.164)	Equator	Yes	Modified Mitutoyo micrometer	Roberts (1997)
	San Diego Bay, California	1991	22	0.149 (0.129–0.180)	Equator	Yes	Modified Mitutoyo micrometer	Roberts (1997)
	San Diego Bay, California	1994	4	0.150 (0.144–0.158)	Equator	Yes	Modified Mitutoyo micrometer	Roberts (1997)
	California	2013	55	0.145 (0.123–0.169)	Equator	Yes	Starrett electronic digital micrometer	Clatterbuck et al. (2018)
	San Francisco Bay, California	2014–2018	332	0.144 ± 0.012*^SD^* (0.109–0.177)	Equator	Yes	Magna‐Mike 8600 HETG	Present study
Mallard	Not specified	pre−1964	1	0.286	Not specified	No	Micrometer screw gauge	Tyler (1964)
	San Francisco Bay, California	2014	2	0.339 ± 0.018*^SD^* (0.326–0.352)	Equator	Yes	Magna‐Mike 8600 HETG	Present study
Snowy plover	Pacific Northwest	pre−1947	9	0.146 ± 0.003*^SE^*	Equator	Yes	Micrometer	Henny et al. (1982)
	Oregon	1949–1953	15	0.151 ± 0.002*^SE^*	Equator	Yes	Micrometer	Henny et al. (1982)
	Sand Lake, Oregon	1980	1	0.163	Equator	Yes	Micrometer	Henny et al. (1982)
	Newport Beach, California	1991	3	0.167 (0.165–0.170)	Equator	Yes	Modified Mitutoyo micrometer	Roberts (1997)
	San Diego Bay, California	1993	3	0.155 (0.144–0.171)	Equator	Yes	Modified Mitutoyo micrometer	Roberts (1997)
	San Francisco Bay, California	2014–2018	34	0.165 ± 0.013*^SD^* (0.133–0.191)	Equator	Yes	Magna‐Mike 8600 HETG	Present study
Wood duck	Not specified	Pre‐1964	1	0.243	Not specified	No	Micrometer screw gauge	Tyler (1964)
	Not specified	Pre‐1982	5	0.300	Equator	Yes	Micrometer	Rahn et al. (1982)
	Wisconsin	Pre‐1986	≥8	0.255 ± 0.004*^SE^* (0.196–0.300)	Shell fragments	Not specified	Tubular micrometer	Soulliere (1987)
	Nevada	2014	39	0.326 ± 0.023*^SD^* (0.273–0.377)	Equator	Yes	Magna‐Mike 8600 HETG	Present study

Note

Means are assumed to be arithmetic unless otherwise indicated. Standard deviation (*SD*) standard error (*SE*) or confidence limit (CL) is indicated using a superscript after the value. Eggs for multiple studies were salvaged and collected from locations known to contain contaminants that could influence eggshell thickness. Most studies that included museum specimens from prior to 1947 state that the researchers compared eggshell thickness between pre‐DDT eggshells from museums and post‐DDT eggshells that were measured at the egg equator. Although most blow‐out holes in museum eggshells are at or near the equator, if eggs from museum collections were measured closer to the blunt end of the egg than the true egg equator it would result in a thinner eggshell measurement (Santolo, 2018).

^a^ Pre‐DDT is put for one study where there were no corresponding dates reported.

^b^ Eggs from Texas and other southern latitudes were used when possible but it is unclear exactly where the museum eggs were collected.

^c^ Measurements may be from the same eggshells.

^d^ Double‐crested cormorant include *P. a. albociliatus* and *P. a. auratus* subspecies.

^e^ Least tern include interior (*S. a. athalassos*), eastern (*S. a. antillarum*), and Pacific coast (*S. a. browni*) subspecies.

Eggshells were typically thickest at the equator and middle portions of the egg and eggshell thickness declined toward each pole, which is consistent with most previous studies (Longcore, Samson, & Whittendale, 1971; Maurer et al., 2012; Orłowski, Siekiera, et al., 2019; Tyler, 1964). Orłowski, Siekiera, et al. (2019) found that the mean eggshell thickness of white stork (*Ciconia ciconia*) eggs was 4.6% and 7.7% thicker at the equator than at either the sharp pole or blunt pole, respectively. Conversely, a study on Eurasian reed warblers (*Acrocephalus scirpaceus*) found the blunt end of the egg to be thicker than the equator (Orłowski et al., 2016), as did a study on domestic Peking duck (*A. platyrhynchos* f. *dom*) (Balkan et al., 2006), domestic guinea fowl (*Numida meleagris galeata*) (Ancel & Girard, 1992), and domestic Japanese quail (*Coturnix japonica*) (Kul & Seker, 2004). The slope of 0.95 we observed for the relationship between eggshell thickness at the sharp pole and the equator indicates that the eggshell thickness at the sharp pole is consistently 95% of the eggshell thickness at the equator. Therefore, when a whole egg is not available and only eggshell fragments can be collected, if the sharp pole can be identified and measured, this relationship may be used to estimate eggshell thickness at the equator.

Physical characteristics of eggs and biological attributes of the clutch may explain variability in eggshell thickness within species. Our use of a minimum eggshell thickness measurement would likely capture the more pigmented and sometimes thinner sections of an eggshell (Gosler et al., 2005), although variation in eggshell thickness with pigmentation has not been supported in all studies (Maurer, Portugal, Boomer, & Cassey, 2011), suggesting that variation in pigmentation within and among some species may complicate interpretations of eggshell thickness. Additionally, the laying order of a clutch may influence the length and width of an egg (Ackerman, Eagles‐Smith, Herzog, Yee, & Hartman, 2016; Penland, 1981); consequently, this may explain some of the observed variability in eggshell thickness within species. Laying order influenced eggshell thickness in falcons (*Falco* sp.), with the thickest eggshells observed for the first egg laid (Castilla, Herrel, et al., 2010), but a study on passerines (Passeriformes) found no such relationship (Orłowski et al., 2016). In addition, other studies indicate that the overall clutch size influences an individual egg's eggshell thickness, with bigger clutches associated with thinner individual eggshells in passerine birds (Orłowski et al., 2016).

We did not detect a decrease in the average eggshell thickness at the equator or sharp pole of the egg with embryonic development, and we found some support for a small increase in thickness of the eggshell membrane and eggshell when all embryo ages were included. Most studies observed eggshell thinning as a result of advances in embryonic development, when the eggshell membrane was removed from the calcite eggshell (Ancel & Girard, 1992; Castilla, Herrel, et al., 2010; Finnlund et al., 1985; Orłowski & Hałupka, 2015; Santolo, 2018). However, removal of the eggshell membrane can remove some of the mammillary core and result in an underestimate of the eggshell thickness (Simkiss, 1961). Furthermore, eggshell membranes may increase in thickness during embryonic development (Castilla, Van Dongen, et al., 2010; Finnlund et al., 1985). However, when we examined eggshells with embryos older than one week of age, we found no evidence of eggshell thinning with embryonic development and we no longer observed an increase in eggshell and membrane with embryonic age. Of note, only 3.5% of embryos were in the final quarter of development. Detectable eggshell thinning due to embryonic development may not occur until the final quarter of the incubation period, similar to observations in capercaillie (*Tetrao urogallus*) eggs (Orłowski, Merta, et al., 2019).

Organochlorine compounds, such as DDT (dichlorodiphenyltrichloroethane), were widely used beginning in the 1940s and were found to decrease eggshell thickness and affect egg survival (Cooke, 1973; Hickey & Anderson, 1968). It is possible that Hg within the egg itself, which also indicates Hg within the female developing the egg (Ackerman et al., 2020), may also influence eggshell thickness, but studies are less conclusive for this contaminant. We found that eggshell thickness, measured either at the equator or the sharp pole, was not correlated with egg content THg concentrations. In contrast, eggshells of Japanese quail thinned significantly when fed diets containing known quantities of mercuric chloride (Stoewsand et al., 1971). Additionally, research on mallard eggshells (Heinz, 1979) showed that eggs of game farm mallards fed a diet of 0.5 ppm Hg had slightly thinner eggshells at the equator (6.6%) than mallards fed on a control diet, but this occurred only in the third generation of mallards fed Hg and not in the first two generations fed Hg (Heinz, 1979). Other field studies have indicated more ambiguous results. Previous research on clapper rail (*Rallus longitrostris*) indicated that eggshells were thinner at a contaminated site than a control site, with Hg the only metal that significantly differed by site (Rodriguez‐Navarro et al., 2002). However, when data were analyzed by site there was no detectable relationship between eggshell thickness and Hg concentrations; consequently, the observed variation in eggshell thickness may have been driven by site characteristics rather than Hg (Rodriguez‐Navarro et al., 2002). Additionally, eggshell thickness and mercury concentrations were not related in eggs of Forster's tern (*n* = 79), black skimmer (*n* = 41) (King et al., 1991), or several shorebird species (Hargreaves et al., 2011).

Eggshell thickness was not related to egg status in the subset of the three avian species where we monitored nests weekly (American avocet, black‐necked stilt, and Forster's tern), similar to previous research on least tern (Koenen & Leslie, 1996). Specifically, eggshells from eggs that did not hatch, either from clutches where no eggs hatched (dead egg status) or from clutches where other eggs in the clutch hatched (failed‐to‐hatch egg status), were not thicker or thinner, on average, than eggs that were progressing normally.

Accounting for eggshell thickness can be important in the accurate calculation of contaminant concentrations in avian eggs. Specifically, using estimates of eggshell thickness results in more accurate (6%–13%) fresh wet weight egg contaminant concentrations than when the eggshell is ignored (Herzog et al., 2016). Using measured eggshell thickness will provide the most accurate calculation of fresh wet weight contaminant concentrations for an individual egg. However, measuring eggshell thickness for every egg can be time consuming and costly. If eggshell thickness cannot be measured for individual eggs, we suggest using mean eggshell thickness for the species (Table 2) in the calculation of fresh wet weight contaminant concentrations instead of using multispecies allometric equations to estimate eggshell thickness. For species where mean eggshell thicknesses do not exist in the literature, you could estimate a species mean equator eggshell thickness using the equation with species mean female body mass in the present study (equator eggshell thickness [mm] = (0.15918 × log_10_(bird body mass) [mg]) – 0.12689), or other available multispecies equations based on other egg morphometrics (Birchard & Deeming, 2009; Maurer et al., 2012). However, individual species can deviate substantially from expected eggshell thicknesses. For example, in the present study, the mean equator eggshell thickness of snowy plover was 26.0% thicker than would be predicted based on the among‐species equation for body mass. Therefore, using multispecies equations to estimate eggshell thickness is a tool that should be employed with caution only in the absence of empirically measured eggshell thickness data for the specific species being studied.

## CONFLICT OF INTERESTS

The authors declare no conflicts of interest.

## AUTHOR CONTRIBUTION

**Sarah H. Peterson:** Conceptualization (equal); Data curation (lead); Formal analysis (lead); Funding acquisition (supporting); Investigation (lead); Methodology (equal); Project administration (supporting); Supervision (equal); Validation (equal); Visualization (lead); Writing‐original draft (lead). **Joshua T. Ackerman:** Conceptualization (equal); Data curation (supporting); Formal analysis (supporting); Funding acquisition (lead); Investigation (equal); Methodology (equal); Project administration (equal); Supervision (equal); Validation (equal); Visualization (supporting); Writing‐review & editing (equal). **Mark P. Herzog:** Conceptualization (equal); Data curation (supporting); Formal analysis (supporting); Funding acquisition (supporting); Investigation (equal); Methodology (equal); Project administration (supporting); Supervision (equal); Validation (equal); Visualization (supporting); Writing‐review & editing (equal). **Matthew S. Toney:** Conceptualization (supporting); Data curation (supporting); Investigation (supporting); Methodology (supporting); Writing‐review & editing (supporting). **Breanne Cooney:** Conceptualization (supporting); Data curation (supporting); Investigation (supporting); Methodology (supporting); Writing‐review & editing (supporting). **C. Alex Hartman:** Conceptualization (supporting); Data curation (supporting); Formal analysis (supporting); Funding acquisition (supporting); Investigation (equal); Methodology (equal); Project administration (supporting); Supervision (supporting); Validation (supporting); Visualization (supporting); Writing‐review & editing (equal). 

## Ethics statement

Research was conducted with the approval of the U.S. Geological Survey Western Ecological Research Center's Animal Care and Use Committee.

## Data Availability

The data in this article are in ScienceBase: https://doi.org/10.5066/P981OW6T.
